# A Comparative Review and Benchmarking Evaluation of Step Detection Methods for Longitudinal Gait Assessment

**DOI:** 10.3390/s26206582

**Published:** 2026-10-16

**Authors:** Alejandro Ena, Jose Javier Serrano Olmedo, Claudia Mazzà, Shibeshih Belachew, Óscar Reyes

**Affiliations:** 1 Indivi Technologies SL, 14001 Córdoba, Spain; alejandro.ena@indivi.io; 2 Center for Biomedical Technology, Universidad Politécnica de Madrid, 28223 Madrid, Spain; josejavier.serrano@upm.es; 3 Indivi AG, 4051 Basel, Switzerland; claudia.mazza@indivi.io (C.M.); belachew@indivi.io (S.B.)

**Keywords:** step detection methods, gait analysis, inertial measurement units, wearable sensors

## Abstract

Gait analysis based on inertial measurement units (IMUs) enables scalable longitudinal gait assessment, with step detection as a foundational component. However, an understanding of available step detection methods and their comparative performance across sensor placements, clinical populations, and real-world environments remains limited. This paper addresses this gap through a twofold contribution. First, a structured taxonomic review organises the landscape into six families—rule-based heuristics, phase portrait, template matching, time–frequency analysis, adaptive oscillators, and machine learning—characterising the trade-offs of each in terms of noise robustness, generalisability, and computational efficiency. Second, nine algorithms drawn from five of these families, all but phase portrait, are benchmarked across three large datasets encompassing healthy adults and people with mobility-impairing conditions such as Parkinson’s disease and multiple sclerosis, at multiple sensor placements under both controlled laboratory and free-living conditions. Accuracy depended on sensor placement, gait pathology and recording environment: step detection proved reliable in structured laboratory settings across most sensor locations and clinical cohorts, whereas unsupervised free-living monitoring produced a substantial degradation for most algorithms. Over all conditions, the three evaluated time–frequency methods obtained the best average ranks for detection accuracy, step-duration error and cadence error.

## 1. Introduction

Gait analysis is the systematic study of human movement during walking, and gait patterns often reflect an individual’s underlying neurological and physiological condition. Consequently, gait analysis is a fundamental tool in clinical settings and is widely used to assess locomotor functions, track disease progression, and examine the efficacy of an intervention [1].

Traditionally, quantitative gait analysis has relied on highly accurate laboratory-based systems, such as force platforms, instrumented walkways, and optoelectronic motion capture systems. While these gold-standard methods provide accurate data, they can be cost-prohibitive, require specialized infrastructure, and restrict data collection to infrequent, supervised assessments. Furthermore, these in-lab tests typically capture only a limited number of gait cycles, providing a brief “snapshot” of mobility that may fail to reflect an individual’s natural performance in real-world environments [2].

Today, modern smartphones and wearable devices are equipped with various built-in sensors, making them a promising solution for longitudinal motor assessment [2]. These technologies, particularly those incorporating Inertial Measurement Units (IMUs) that integrate accelerometers, gyroscopes, and magnetometers, have gained significant attention as a low-cost, portable, and noninvasive alternative. They make it possible to capture gait data more frequently and unobtrusively in a person’s own home or natural environment. This shift from supervised, in-clinic assessments to unsupervised, real-world data collection is foundational for capturing objective, quantifiable measures of function as it is actually expressed in daily life, complementing rather than replacing the sensor-based assessments already used in the clinic.

Gait is a cyclic movement, and the basic units of walking activity are steps (the interval between the Initial Contact (IC) of one foot and the contralateral foot) and strides (the interval between two subsequent ICs of the same foot). The process of accurately identifying these events—step/stride detection—is therefore the cornerstone of the entire gait analysis pipeline, and the accuracy of all subsequent gait parameters, such as cadence, gait speed, and stride-to-stride variability, is critically dependent on the ability to correctly identify these individual steps [3,4].

However, transferring step detection from the laboratory to unsupervised, real-world environments introduces considerable challenges. First, the accuracy of any algorithm is immediately affected by population variability, as it must be robust not only in healthy controls but also in people with pathological gait, whose movements are often more irregular and produce noisier signals. Second, real-world monitoring typically relies on consumer-grade devices, whose IMUs may have lower signal fidelity than their laboratory-grade counterparts. Third, many algorithms are designed and validated for a specific anatomical sensor placement (e.g., the lower back) and may suffer performance degradation when transferred to alternative locations. This issue is compounded in free-living scenarios where the sensor is unconstrained: a device may be carried loosely in a trouser pocket or waist bag, creating movement artifacts unrelated to gait. Finally, real-world data are not limited to straight, steady-state walking; instead, they contain a complex mix of activities that disrupt clean signal patterns. Transient events such as turning, pausing, navigating uneven terrain, or climbing stairs make robust step detection a primary signal processing challenge [5,6].

A wide variety of IMU-based step detection algorithms have been proposed in the literature [3]. While prior works [3,7] broadly categorised these methods into two main branches—traditional signal-processing (heuristics) and Machine Learning (ML)—the landscape has evolved significantly. Heuristic approaches typically rely on expert-defined, hand-crafted rules, such as identifying local extrema or applying time–frequency analysis. Conversely, the ML category has seen significant growth, ranging from classical models, such as Hidden Markov Models (HMMs) and Support Vector Machines (SVMs) [8], to more recent and powerful Deep Learning (DL) architectures, like Temporal Convolutional and Recurrent Neural Networks [9,10]. However, these categories are not mutually exclusive; it is increasingly common to find hybrid algorithms designed to leverage the computational efficiency of heuristic methods while capitalizing on the robustness of data-driven models.

Despite the wide range of solutions proposed for step detection using IMU sensors, their comparative performance has rarely been evaluated under common conditions, largely owing to the scarcity of suitable shared datasets. Many algorithms have been validated on a single dataset, a single sensor placement, or a single population, resulting in a lack of standardised benchmarks for unsupervised remote gait analysis. Substantial progress towards standardisation has nonetheless been made through two complementary resources: the WearGait-PD reference dataset [11] and the Mobilise-D consortium [5,12,13]. Together, these efforts cover complementary aspects of gait assessment: WearGait-PD supports multi-placement laboratory benchmarking, whereas Mobilise-D combines structured laboratory testing with unsupervised free-living monitoring. Both, however, validate a defined pipeline within a fixed measurement configuration, leaving open how the performance of the different algorithmic families, and their ordering relative to one another, varies with sensor placement, cohort and recording environment.

Consequently, this paper pursues two objectives. First, it presents a structured review of existing IMU-based step detection algorithms, taxonomizing the main methods and highlighting their respective advantages and limitations. Second, it conducts an experimental benchmarking study, evaluating a selected subset of these methods against reference data from three datasets comprising both healthy controls and pathological populations. Rather than aggregating previously published results, a common set of nine algorithms, drawn from five of the six families reviewed here, is evaluated under a single harmonised protocol: the sampling rate, matching tolerance, error metrics and the algorithm parameters are identical throughout. Comparisons therefore differ not only in sensor placement, gait pathology, and recording environment, but also in the acquisition systems, reference standards, and experimental protocols of the original datasets. The stability of the resulting ranking of methods across these varying conditions can thus be quantified rather than assumed. Ultimately, this study elucidates the performance trade-offs of these step detection algorithms, providing empirical evidence to support the selection of appropriate methods for gait assessment in longitudinal monitoring.

## 2. Step Detection Methods

Over the years, the literature has evolved from simple thresholding techniques to complex data-driven models. Building on prior reviews [3,7], we organise existing step detection methods into six primary families based on their underlying processing paradigm: Rule-Based heuristics (Time-Domain), Phase Portrait analysis (Geometric), Template Matching (Pattern Recognition), Time–Frequency analysis (Spectral), Adaptive Oscillators (Dynamical Systems), and Machine Learning (Statistical/Data-Driven). Table 1 provides a comparative summary of these taxonomies, highlighting their specific techniques, strengths, and inherent limitations.

### 2.1. Rule-Based Methods

Rule-based methods represent a deterministic paradigm for step detection, where algorithms are constructed using explicit, pre-defined logical conditions. These methods rely on expert knowledge derived directly from the biomechanics of human locomotion [3], and they fundamentally operate on the assumption that the gait cycle generates a quasi-periodic and repetitive signal signature characterized by distinct features. Consequently, the algorithm’s task is to identify these features within the raw or pre-processed inertial data using fixed or adaptive criteria. In the literature, these heuristics are predominantly implemented through two main techniques: the detection of local signal extrema (peaks and valleys) and the analysis of thresholds as zero-crossings.

Regarding the detection of local extrema, Peak Detection is a very common strategy that identifies key gait events—such as IC or Final Contact (FC)—as distinct local maxima or minima in the inertial signal. Since raw data often contains high-frequency noise that can trigger false positives, low-pass filtering is a standard prerequisite. However, filtering introduces an unavoidable phase lag. Although this delay can be eliminated using zero-phase filtering, this technique requires the complete signal sequence, limiting its application strictly to offline analysis [14,15,16].

In scenarios involving impaired gait or high signal noise, the signal often contains local extrema unrelated to actual gait events, complicating the step detection process. To address this, algorithms employ amplitude thresholds, operating on the hypothesis that artifacts possess distinct signal characteristics compared to true gait events. These thresholding strategies fall into two primary categories: fixed and adaptive. Fixed thresholds are static values defined a priori; while computationally simple, they lack robustness against inter-subject variability, changes in sensor placement, and fluctuations in walking speed. In contrast, adaptive thresholds dynamically adjust based on real-time signal statistics (e.g., the mean or standard deviation over a sliding window). For instance, Jiang et al. (2019) [17] successfully utilized adaptive thresholding to detect gait events across three Parkinson’s disease datasets with varying sensor locations. However, it is worth noting that their validation did not account for variable walking speeds, a critical factor in daily living contexts.

Beyond differentiating true peaks from artifacts, thresholds also serve as the primary detection mechanism in Zero-Crossing algorithms. These methods exploit the oscillatory nature of gait, positing that a complete step cycle involves a periodic reversal of the signal’s sign relative to a mean value (typically zero). A representative implementation is that of Shin and Park (2011) [18], who calculated the norm of the tri-axial acceleration, applied a smoothing window to remove high-frequency jitter, and identified steps by counting the crossings of the mean-centered signal. While they reported high accuracy, it is critical to note that their validation was restricted to a single subject performing repeated trials. Consequently, the generalizability of this approach to diverse populations or pathological gait patterns remains unverified.

The principal advantages of rule-based methods are their ‘white-box’ transparency and computational efficiency. Because their decision logic relies on interpretable biomechanical rules rather than opaque statistical features, they allow researchers to easily diagnose errors, as the cause of a missed step is directly traceable to a specific rule. Furthermore, since they consume minimal processing power, they are ideal candidates for real-time implementation on battery-constrained embedded systems. However, this simplicity comes at the cost of robustness, since these algorithms exhibit high parametric sensitivity; a threshold tuned for a healthy adult on a treadmill often fails when applied to the irregular gait of a person with a neuromusculoskeletal condition in a free-living environment. Consequently, achieving generalization often requires rigid sensor placement or multi-sensor setups to provide necessary context, which limits their utility in unconstrained, single-sensor applications. For these reasons, current literature [3] increasingly positions rule-based methods not as standalone solutions, but as efficient preprocessing stages within hybrid architectures.

### 2.2. Phase Portrait Methods

Phase Portrait methods approach gait analysis by constructing a state-space representation of the movement. Instead of analyzing signals purely in the time domain, these algorithms plot a characteristic orbital trajectory (limit cycle) in the phase plane, typically using a signal and its first derivative as coordinates (e.g., joint angle against angular velocity, or acceleration against velocity).

In the literature, applications of this method vary by how they interpret the trajectory. Salguero et al. (2012) [19] identified specific gait phases as distinct geometric regions within the loop generated by pelvic acceleration and velocity. Conversely, more recent approaches, such as those by Quintero et al. (2017) [20] and Choi et al. (2023) [21], calculate the continuous phase angle and trigger events when this angle reaches specific, pre-determined thresholds known to correlate with initial or final contact.

Phase Portrait methods offer a balance between computational efficiency and information density. As a subset of heuristic algorithms, they share the low computational cost of time-domain methods, making them equally suitable for embedded systems and effective with a single sensor. By exploiting the bivariate nature of the signal, they provide a continuous estimate of gait progression rather than just discrete event detection. Despite these advantages, their robustness is fundamentally constrained by the geometric regularity of the trajectory. These algorithms rely on the assumption that gait forms a stable, quasi-circular limit cycle. However, in real-world scenarios—characterized by transient movements, variable speeds, or pathological irregularities, the orbital trajectory often becomes distorted or fails to form a closed loop. Consequently, when the limit cycle loses its consistency, the validity of the phase estimation is compromised, leading to a significant reduction in step detection accuracy [22].

### 2.3. Template Matching Methods

Unlike rule-based methods that search for specific local features, Template Matching algorithms operate on a pattern recognition paradigm, treating the gait cycle as a holistic waveform. The fundamental premise is that walking generates a repetitive signal signature. Consequently, these algorithms generally comprise two distinct phases: the generation of a representative reference pattern (the ‘template’) and the continuous evaluation of the incoming signal against this prototype using a similarity metric.

The first phase, template generation, is critical for defining the algorithm’s target. These references can be constructed manually through expert annotation, as demonstrated by Oudre et al. (2018) [23], who compiled a comprehensive library of gait templates from annotated datasets. Alternatively, a more automated approach involves averaging multiple valid gait cycles extracted from a reference ground truth to create a mean subject-specific prototype [24]. Once the template is defined, the algorithm scans the input stream to quantify similarity. Early implementations relied on metrics such as Euclidean distance [25] or cross-correlation [26]. While computationally efficient, these deterministic metrics suffer from a significant limitation: they strictly require the input signal and the template to share identical lengths and shapes. This rigidity renders them fragile in real-world scenarios, where natural fluctuations in walking speed constantly alter the duration of the stride cycle. In addition, because a template encodes a specific gait morphology, references built for one individual or population cannot be directly reused for others.

To address the limitations of linear matching, the literature has widely adopted Dynamic Time Warping (DTW) [27]. DTW is a dynamic programming algorithm that finds the optimal non-linear alignment between two time sequences by locally stretching or compressing the time axis, subject to monotonicity, continuity, and boundary constraints. This allows the template to be effectively stretched or compressed to match the input signal, providing robustness against temporal variations. The versatility of this approach extends beyond simple step detection to the identification of pathology. For instance, Xu et al. (2017) [28] utilized DTW to detect Freezing of Gait in people with Parkinson’s disease. By cross-referencing real-time sensor data against normal-walking and freezing-gait templates, they achieved detection accuracies exceeding 90%, demonstrating the method’s ability to discriminate between complex functional states.

In summary, Template Matching methods offer a highly personalized approach to gait analysis that excels in handling morphological variability. Particularly when paired with DTW, they can achieve high accuracy across varying walking speeds without the phase lag issues associated with frequency filtering. However, these methods are computationally intensive compared to rule-based methods, which can limit their deployment in real-time applications on low-power embedded hardware. Furthermore, they suffer from a “cold start” dependency, where the system requires a high-quality, representative template to function. Because gait morphology varies substantially across individuals and clinical populations, a template built from one cohort cannot be assumed to generalise to another; if the template is not representative of the user’s current condition, or if valid strides cannot be pre-segmented to build it, the step detection mechanism is compromised. Finally, similar to heuristic approaches, they remain dependent on empirically tuned similarity thresholds to distinguish valid gait events from motion artifacts or spurious signal fluctuations.

### 2.4. Frequency Methods

Frequency-based methods operate on the fundamental hypothesis that human gait is a quasi-periodic phenomenon. Consequently, inertial signals should contain distinct, repetitive frequency signatures corresponding to the stride cycle that can be separated from noise or harmonics. Traditional spectral techniques, such as the Fast Fourier Transform (FFT), utilize this periodicity to identify dominant gait frequencies. Approaches like those by Kang et al. (2018) [29] and Werner et al. (2021) [30] demonstrate this by filtering signals around the fundamental step frequency to isolate valid heel strikes. However, these methods rely on a strict assumption of stationarity, i.e., the frequency content of the signal remains constant over time. In real-world environments, gait is inherently transient; speed fluctuates, and events like heel strikes are impulsive rather than continuous. Because the FFT aggregates frequency information over the entire signal duration, it obscures the temporal location of these events, rendering it ill-suited for precise event detection [4].

To address non-stationarity, early solutions employed the Short-Time Fourier Transform (STFT), which analyzes the signal in fixed time windows. While this provides some temporal localization, it is fundamentally constrained by the Heisenberg-Gabor uncertainty principle: a narrow window offers good time resolution but poor frequency resolution, while a wide window improves spectral detail at the cost of temporal blurring [31]. Furthermore, by relying on a fixed window size, the STFT assumes that the signal is stationary within that specific analysis window. Cohen (1995) [32] corroborated that the uncertainty principle prevents the simultaneous precise localization of high and low-frequency components, which is essential for correctly characterizing the variability of the gait pattern.

Research interest has consequently turned to multiscale analysis techniques, specifically the Wavelet Transform (WT), which has proven superior to traditional spectral methods for analyzing non-stationary kinematic data [4]. Unlike the STFT, which relies on a fixed window, the WT employs an adjustable window size that naturally adapts to the spectral characteristics of the signal. The fundamental principle involves decomposing the signal using a Mother Wavelet function that can be scaled in frequency (dilated or compressed) and shifted in time. This capability, known as Multiresolution Analysis (MRA), allows the uncertainty principle to be managed dynamically by using long time intervals for precise low-frequency information (gross movement) and short time intervals for high-frequency transients (heel strikes).

Wavelet methods are predominantly classified based on their discretization strategy into three categories: Discrete, Continuous, and Empirical. The Discrete Wavelet Transform (DWT) represents the most computationally efficient implementation. It decomposes the signal into approximation coefficients (low frequency) and detail coefficients (high frequency) using Mallat’s Pyramidal Algorithm [33] through a series of Low-Pass and High-Pass filter banks. This hierarchical decomposition allows researchers to isolate specific frequency bands related to gait while discarding noise-related coefficients. For instance, Livolsi et al. (2021) [34] combined DWT with adaptive oscillators to achieve robust detection by filtering the hip angle signal. Similarly, Navarro et al. (2024) [35] utilized Daubechies and Symlets wavelets to decompose and reconstruct angular velocity signals from IMUs, effectively eliminating noise to detect IC and FC events via simple thresholding. While DWT is highly efficient for general feature extraction, its discrete nature limits its resolution for precise temporal localization compared to continuous methods.

The Continuous Wavelet Transform (CWT), conversely, prioritizes resolution over computational efficiency. By calculating the correlation between the signal and the Mother Wavelet at all possible scales and positions without downsampling, CWT generates a highly detailed Time–Frequency Representation (TFR). This is particularly advantageous for identifying subtle transients and abrupt changes in the signal. Khandelwal and Wickström (2018) [36] exploited this by tracking dominant spectral energy scales across different body locations to estimate ICs. Likewise, McCamley et al. (2012) [37] applied Gaussian wavelets to smooth and differentiate vertical acceleration signals, identifying events at local minima and maxima. This foundational approach has been further refined in subsequent literature. Bourke et al. (2020) [38] and Ionescu et al. (2020) [6] improved step detection robustness by incorporating specific pre-processing steps and optimizing wavelet scale selection to better match human gait characteristics. More recently, Angelini et al. (2025) [39] introduced an adaptive framework that dynamically adjusts wavelet scales to the instantaneous walking frequency, significantly enhancing performance in variable gait conditions.

Furthermore, CWT is not utilized solely for signal denoising and reconstruction; it is increasingly employed to directly analyze the frequency composition of gait over time through TFRs. While standard TFRs can suffer from spectral smearing or limited resolution due to the uncertainty principle, advanced post-processing techniques like Synchrosqueezing [40] have been developed to reassign energy to the true instantaneous frequencies, sharpening the representation. Ena et al. (2024, 2026) [41,42] demonstrated the efficacy of these post-processed TFR methods, achieving a significant improvement in event detection accuracy by effectively building a ridge that denotes the evolution of the fundamental frequency along the time.

Finally, the Empirical Wavelet Transform (EWT) [43] represents an adaptive paradigm that constructs custom filter banks based on the specific Fourier spectrum of the input signal, rather than using a pre-defined basis function. This allows for the extraction of interpretable modes directly related to the signal structure. While its application to event detection is limited, Ahmed et al. (2021) [44] demonstrated its potential by successfully identifying complex pathological states like Freezing of Gait in people with Parkinson’s disease, suggesting it may offer superior adaptability for abnormal gait patterns.

In summary, time–frequency methods offer a powerful compromise between the temporal precision of peak detection and the noise robustness of spectral analysis. Their primary advantage is the ability to perform denoising and feature extraction simultaneously through Multiresolution Analysis. However, this sophistication introduces complexity, since performance is highly sensitive to the choice of the Mother Wavelet; selecting a basis function that does not match the morphological shape of the user’s gait signal can lead to poor correlation. Furthermore, boundary effects at the edges of the analysis window can introduce artifacts, and the computational demand—particularly for CWT—remains a barrier for ultra-low-power real-time implementation compared to simple time-domain heuristics.

### 2.5. Adaptive Oscillators

Adaptive Oscillators (AO) are dynamical systems designed to synchronize with periodic or quasi-periodic signals without requiring complex pre-processing. Introduced to the gait field by Righetti et al. (2009) [45], AO essentially function as a non-linear filter that learns the fundamental frequency (ω) and instantaneous phase (ϕ) of an input signal in real time. Unlike static filters, the AO dynamically adjusts its internal state variables to follow the evolution of the input, allowing it to provide a smooth, continuous estimate of the gait cycle phase (0−2π) even as the user’s cadence varies. Because they operate on the general principle of periodicity, they are highly versatile and can be applied to various sensor modalities, including gyroscopes, accelerometers, and electromyography [46].

Recent literature has focused on optimizing the computational efficiency and phase linearity of these systems. You et al. (2024) [47] achieved execution times of less than 1 ms per sample on standard embedded hardware. To address the issue of signal distortion, Yan et al. (2017) [48] proposed a mechanism to linearize the estimated phase, ensuring it increases monotonically from 0 to 2π while employing error compensation to prevent abrupt phase jumps at the transition between strides. The importance of this linearization is associated with the fact that it allows gait events to be detected by simply checking if the phase angle has reached a specific threshold, providing a detection mechanism that is much more stable than raw signal thresholding.

In summary, Adaptive Oscillators offer a compelling alternative for continuous phase estimation, particularly in low-power embedded hardware. Their primary strength is their intrinsic noise robustness, associated with the fact that the oscillator possesses its own inertia and it naturally filters out high-frequency disturbances without the lag associated with low-pass filtering. However, AOs suffer from a “convergence latency”—a period required to synchronize with the signal. Standard AOs struggle with non-stationary conditions, such as gait initiation and termination, often requiring several steps to lock onto the correct frequency. While recent optimizations like those by Wang et al. (2024) [49] have reduced this synchronization period from 4 strides to approximately 2.6, this delay remains problematic for real-time detection, as it implies that the first few steps of any walking bout may be missed. Furthermore, performance degrades in asymmetric or pathological gait [50], and the system requires careful tuning of internal coupling constants, creating a difficult trade-off between fast learning (reactivity) and stability (noise rejection).

### 2.6. Machine Learning Models

Machine Learning (ML) approaches represent a paradigm shift from deterministic rule-based algorithms. Instead of relying on rigid biomechanical rules, ML methods formulate gait detection as a statistical pattern recognition problem. These techniques can be generally categorized based on how they process information: Sequential Probabilistic models, which explicitly model the temporal evolution of gait; Feature-Based Discriminative models, which classify segments based on handcrafted characteristics; and Deep Learning (DL), which automates feature extraction through hierarchical neural networks.

Sequential Probabilistic Models seek to exploit the cyclical nature of walking by explicitly modeling the probability of transitioning between specific gait phases. Among these, HMMs are the most prevalent [3]. HMMs operate on the premise that the observable sensor data is generated by a sequence of “hidden” states corresponding to gait events. This structure allows them to incorporate temporal logic directly into the detection process. However, standard HMMs typically assume fixed transition probabilities, limiting their flexibility in dynamic scenarios where gait patterns change rapidly [51]. Furthermore, they are rarely validated on non-standard sensor locations, with the exception of works like Garcia et al. (2022) [52], who successfully implemented an HMM on a smartphone in a trouser pocket, albeit in the laboratory and under specific conditions.

Feature-Based Discriminative Models, such as SVMs, K-Nearest Neighbors (K-NN), and Random Forests (RF), focus on establishing optimal decision boundaries between gait phases. The primary characteristic of this group is their reliance on handcrafted features (e.g., mean, standard deviation, entropy) that must be computed beforehand for each time window. SVMs, in particular, are favored for their rigorous theoretical foundation and their ability to generalize well on limited datasets [53,54]. However, this dependence on manual feature engineering is a significant limitation, since determining the most relevant features is often non-obvious. Furthermore, unlike sequential models, these classifiers typically treat each time window as an isolated observation, ignoring the historical context and sequential dependencies inherent in the gait cycle.

Deep Learning (DL) overcomes the limitations of manual feature engineering by utilizing multi-layered neural networks to automatically learn hierarchical representations from raw inertial data [55]. Architectures typically include Convolutional Neural Networks (CNN), which are particularly robust at extracting local spatial features for event localization [10], and Temporal CNNs or Recurrent Neural Networks (RNN)—specifically Long Short-Term Memory (LSTM) networks—which are designed to capture the temporal order that standard discriminative models miss [9]. More recently, transformer-based models [56] and selective state-space models [57] have been applied to gait analysis, demonstrating accurate modeling of long inertial-data sequences. However, their applications have primarily focused on activity recognition [56], gait identity recognition [58], and the detection of clinical events such as Freezing of Gait [59], rather than on the localization of individual gait events.

In summary, Machine Learning can offer superior robustness compared to heuristic methods, particularly in pathological populations. Deep Learning models, specifically, have demonstrated the highest reliability in signal segmentation due to their generalization capabilities [55]. However, this performance comes with significant barriers. First, these models are “data-hungry”, requiring massive labeled datasets to avoid overfitting. Second, they are computationally intensive; running a deep model inference on a low-cost sensor can often be unfeasible compared to a simple peak detector. Finally, they can suffer from variability in performance when encountering subjects never seen during training [60], so the robustness they offer in pathological populations extends only to the gait patterns their training set contained, and their black-box nature makes it difficult to diagnose why specific step detections fail.

## 3. Benchmark Comparison of Step Detection Methods

While the previous section provided a theoretical taxonomy of existing step detection paradigms, assessing the real-world suitability and accuracy of step detection methods requires rigorous empirical validation. Many algorithms achieve high accuracy in their original publications, yet they are frequently evaluated on disparate datasets, at varying sensor locations, and often in healthy populations only. Notable efforts toward standardisation have been made, most prominently by the Mobilise-D consortium [5,12,13] and by work built on the WearGait-PD dataset [11], which established validated pipelines and reference protocols for real-world gait assessment. The Mobilise-D technical validation [12], for instance, compared four initial contact detection algorithms from a single lower-back placement, and the WearGait-PD work [11] released a reference dataset recorded at a fixed set of body-worn placements under structured walking protocols. Because these efforts were directed at specific algorithmic pipelines and a limited set of sensor placements, a head-to-head comparison of diverse step detection families across multiple sensor placements, clinical populations and recording environments is still lacking.

This section presents a benchmark analysis of nine step detection algorithms, evaluated under a single harmonised protocol across three large datasets. In addition to sensor placement, gait pathology, and recording environment, the datasets differ in acquisition system, reference standard, and experimental protocol. All algorithms were then evaluated on the same recordings within each dataset, ensuring that they shared these dataset-specific conditions in each comparison. The following subsections detail the specific algorithms selected from the taxonomy, the datasets used for assessing step detection, and the statistical metrics employed to quantify step detection accuracy. Subsequently, the empirical results of this comparative analysis are shown, followed by a detailed discussion interpreting the findings to guide the selection of appropriate methods for remote gait assessment.

### 3.1. Selected Step Detection Algorithms

Based on the theoretical taxonomy presented in Section 2, a representative subset of step detection algorithms was selected for experimental benchmarking. Open-source availability (to ensure reproducibility) and methodological diversity (covering distinct paradigms) were prioritized as the primary selection criteria. However, in cases where an open-source implementation was unavailable, the provision of sufficient methodological details to enable independent replication was strictly required. Each selected method was assigned a unique alphanumeric identifier, as summarized in Table 2.

In total, nine step-detection methods were selected for the benchmark comparison: three rule-based methods, three frequency-based methods, one template-matching method, one adaptive oscillator, and one machine learning model. Five of the six families reviewed in Section 2 are therefore represented experimentally. The algorithms were implemented as described in each relevant publication, without further tuning of their parameters. The machine learning model [10] was applied by using the pre-trained weights and peak-detection parameters reported by the authors. Evaluating the machine learning model in its pre-trained version allowed to test the ability of a data-driven step detector to generalise to previously unseen sensor placements, populations and recording conditions.

Finally, no phase-portrait method was included in the empirical evaluation. The algorithms identified in that family are formulated for continuous gait-phase estimation from joint kinematic signals (such as hip or thigh angle, or pelvic acceleration and velocity) in the context of exoskeleton and rehabilitation-robot control, and they require a segment-specific sensor with known orientation, or a derived joint angle, as input. They are consequently not transferable to the placement-agnostic, single-IMU evaluation protocol assessed in this work.

*Rule-Based methods.* The method proposed by Shin and Park (2011) [18] (RB1) was selected for the benchmark. The second rule-based method selected was the one developed by Lee et al. (2010) [61] (RB2), where a morphological filter—comprising erosion, dilation, opening, and closing operations—is applied alongside a Least-Squares Acceleration Filter to amplify signal peaks prior to ICs detection.

In addition to these two rule-based methods, the algorithm proposed by Benson et al. (2019) [62] (RB3) was adapted directly from the algorithm described in the authors’ publication. Although originally conceived for running analysis, it is hypothesized that its architecture—designed to manage high biomechanical variability—offers sufficient robustness for unconstrained walking, where search windows are defined based on vertical acceleration peaks, and ICs detection is refined through a hierarchical classification of peaks within the anteroposterior acceleration signal.

*Frequency-Based Approaches.* The foundational frequency-based algorithm proposed by McCamley et al. (2012) [37] (FB1) was evaluated, where a CWT is leveraged to process the vertical acceleration signal by utilizing a Gaussian mother wavelet at an approximate gait frequency scale, allowing ICs to be identified directly from the local minima in the signal reconstructed from the obtained coefficients. The adaptation proposed by Paraschiv-Ionescu et al. (2020) [6] (FB2) was also selected, where the vertical acceleration signal is first bandpass-filtered (0.15–3.14 Hz) and integrated via cumulative trapezoidal integration. Subsequently, a Gaussian wavelet is applied to simultaneously smooth and differentiate the integrated signal, enabling the identification of ICs as local minima situated between specific zero-crossings.

In addition, the FAIR-Q (Frequency-Adaptive Iterative and Robust Quantification) algorithm, proposed by Ena et al. (2024, 2026) [41,42] (FB3), was incorporated to the benchmark comparison. Although an open-source implementation of FAIR-Q is not publicly available, sufficient methodological details are reported by the authors to enable its replicable implementation. In this approach, a high-resolution TFR of the vertical acceleration, generated via the Synchrosqueezing technique, is utilized to estimate the instantaneous gait frequency and extract a spectral ridge. Subsequently, the vertical acceleration signal is segmented into stationary periods based on abrupt ridge changes, and a dynamic passband filter is designed to optimize the signal-to-noise ratio. Finally, ICs are detected as local minima within the filtered signal.

*Template matching.* The algorithm proposed by Filippou et al. (2023) [63] (TM1) was included. In this method, a customized template matching strategy is employed to mitigate gait quantification errors in populations with biomechanical irregularities, where a subject-specific template is constructed that preserves signal morphology while adapting to phase and frequency variations. A 3Hz low-pass filter is applied, and step events are subsequently detected by comparing the vertical acceleration signal to the reference template using DTW, and utilizing a similarity threshold for final ICs classification.
*Adaptive Oscillators.* The method proposed by Tomc and Matjačić (2023) [50] (AO1) was evaluated. Although originally validated using a head-mounted IMU, the algorithm is mathematically formulated for any continuous oscillatory movement, thereby permitting its adaptation to alternative sensor placements. A bank of Adaptive Oscillators is applied to the vertical acceleration signal, whereby its frequency and phase are learned without the latency typically associated with low-pass filtering. The dynamic evolution of the oscillators’ states is governed by periodically detecting peaks within a sliding window, ensuring the system’s estimations are continuously aligned with actual gait events. Ultimately, ICs are identified through peak detection within the generated phase signal.
*Machine Learning.* The deep learning model proposed by Romijnders et al. (2022) [10] (ML1) was included as the only publicly available model identified in the literature whose released pre-trained weights had been trained directly for the detection of initial contacts. In this method, a Temporal Convolutional Network maps the triaxial acceleration and angular velocity of a single IMU to a per-sample likelihood of an initial and a final contact. The network was trained on recordings from shank-mounted sensors, and learned to detect only the initial and final contacts of the leg on which the sensor was worn, rather than those of both legs.

### 3.2. Benchmark Datasets

Data included in the benchmark comparison were derived from three cross-sectional studies, which were specifically selected to evaluate algorithmic robustness against primary sources of variability in gait analysis: pathological gait impairments, sensor placement, and recording conditions ranging from controlled laboratory settings to unconstrained free-living environments. Furthermore, synchronized reference systems were utilized across all datasets to detect gait events, thereby enabling the objective quantification of estimation errors of the selected step detection algorithms.

A comprehensive technical verification was conducted to assess data quality and finalize the selection of test cases. Acceleration signals were temporally aligned where necessary, and systematically evaluated for missing values, sufficient duration, and sampling frequency stability. To enable a fair comparison across the heterogeneous acquisition systems, all signals were harmonised to a common sampling rate of 50 Hz, matching the lowest native sampling frequency among the included datasets. This rate preserves the relevant gait-event dynamics, as the frequency content of locomotion-related acceleration lies well below the corresponding 25 Hz Nyquist limit. A minimum signal duration threshold of 10 s was established, mainly due to initialization requirements of specific step detection algorithms. For instance, the adaptive oscillator-based method (AO1) [50] requires sufficient time to achieve phase synchronization with the input signal, while the template-matching method (TM1) [63] necessitates an adequate initial data buffer to construct an individualized reference template.

Ground-truth gait events were filtered to exclude foot contacts occurring during turns, which were identified using an adapted version of the El-Gohary algorithm [64]; this algorithm detects turns from the angular velocity around the vertical axis of the lower-back IMU. Only foot contacts occurring during straight-line walking segments were retained. This approach is consistent with previous gait-event validation studies in which straight-line walking and turning are treated as distinct conditions [65]. The resulting turn windows were then also applied to the outputs of the step-detection algorithms: each method was run on the complete recording, including turning periods, and detections falling within a turn window were subsequently excluded from the evaluation. Thus, the same turn windows were used to filter both ground-truth events and algorithm detections, ensuring that both were evaluated over the same walking segments. This procedure restricted the performance evaluation to uninterrupted straight-line walking and minimized the potential confounding effect of the kinematic variability associated with turning.

*CorLab dataset.* This dataset includes data derived from 83 healthy controls -HCs- (ethical approval 064650 from the University of Sheffield, UK). The participants (demographic data in Table 3) performed three repetitions of two supervised, structured tests on a flat indoor surface:
  - 30-s walk test: Participants walked a curvilinear path within a 5-m-sided square room, and they were instructed to walk at their self-selected pace without stopping or interruptions, and to avoid abrupt turns as much as possible.
  - 30-s U-Turn test: Participants walked 3 m in one direction, performed a 180-degree turn, and walked again in the opposite direction. This continuous sequence was repeated for 30 s.

During these tests, inertial sensor data were simultaneously collected by a reference system (Awinda, Xsens [66], sampling frequency 60 Hz) and smartphones (sampling frequency 50 Hz). The Xsens reference system comprised 17 IMUs, each equipped with 3D accelerometers, gyroscopes, and magnetometers. These sensors were attached to specific body segments -head, shoulders, upper arms, forearms, hands, upper legs, lower legs, and feet- using straps, providing the data required to reconstruct full-body 3D kinematics. Gait events were derived from the lower-limb sensors by processing the foot-contact information provided by the Xsens system. Additionally, one IMU was rigidly attached to the back of a smartphone for synchronization and alignment purposes.

The tests were performed with each participant wearing a pair of smartphone (one Android and one iPhone), whose locations were selected from five possible placement conditions: left/right front pockets, left/right back pockets, and an anteriorly located waist-worn pouch. The assignment of Android and iPhone devices to the left or right side was randomized across participants but stratified at the group level to ensure a balanced number of recordings for the left and right legs. Finally, the smartphones were randomly selected from a pool of 10 Android devices (from 7 different manufacturers, Android versions ≥9 and ≤12) and 7 different iPhone models (iOS versions ≥15.7 and ≤16.4.1).

Following technical verification, the final dataset for analysis consisted of 493 30-s walk trials and 489 30-s U-Turn trials. With participants equipped with both a reference system and two smartphone devices, data were analyzed from: (1) XSens IMU sensors located on the head, lower back, left/right mid-upper legs, left/right forearms, and left/right hands; (2) smartphones located on left/right front pockets, left/right back pockets, and an anterior waist-worn pouch.

*WearGait-PD dataset.* This dataset [11] comprised data collected across three clinical sites, involving 85 HCs and 100 people with Parkinson’s disease (PD) (see demographics in Table 3), and the data were collected across three hospitals. Participants performed the following six structured tests:
  - Off-Mat test: Participants started approximately 5 feet off a mat, walked across the mat and approximately 5 feet off the other side of the mat. Participants turned around off the mat and repeated this pass until a total of 4 passes were made. This trial was performed twice at two speeds: self-selected pace and hurried pace.
  - On-Mat test: Participants started on the mat, walked across the mat, stopped, and turned around at the other end of the mat, remanining on the mat the entire time. Participants repeated this until a total of 4 passes were made. This trial was performed twice at two speeds: self-selected pace and hurried pace.
  - Self-pace on-mat test: Participants repeated the self-pace on-mat walking task as described above but were instructed to alternate between left and right turns.
  - Self-pace doorpat test: A mock door frame approximately 3 ft × 8 ft was placed across the center of the mat over a pattern of lines perpendicular to the direction of travel. Participants repeated the self-pace off-mat walking task as described above by walking across the mat through the mock door frame.

During all tests, participants were equipped with a configuration of XSens system which comprised 13 IMU sensors (Xsens MTw Awinda, original sampled at 1000 Hz and subsequently downsampled to 100 Hz). These IMU sensors were attached to specific body segments: head, xiphoid, lower back, wrists, upper legs, shanks, ankles, and feet. Gait events were derived from a 16-foot × 2-foot ProtoKinetics Zeno Walkway Gait Analysis System [67], which detected pressure data and foot contacts.

Following technical verification, the final dataset for analysis consisted of 415 trials for HC and 490 for PD participants. With participants equipped with the XSens system, data were analyzed from sensors located on the head, lower back, left/right mid-upper legs, and left/right forearms (wrists). For the selected trials, only the foot contacts detected by the walkway that occurred outside of abrupt turns were analyzed.

*Mobilise-D dataset.* This dataset derived from the Mobilise-D project [12,13], an initiative that produced validated and accepted digital mobility outcomes to monitor the daily life gait of individuals with various mobility impairments. This dataset includes a total of 108 participants (demographic data in Table 3), comprising 20 HCs, 20 PD, 20 people with Multiple Sclerosis (MS), 19 people recovering from a Proximal Femoral Fracture (PFF), 17 people with Chronic Obstructive Pulmonary Disease (COPD), and 12 people with Congestive Heart Failure (CHF). The participants performed six structured walking tests:
  - Straight walking test: Participants were asked to walk a predefined path of 5 m from a standing start to a standing end. This trial was performed twice at three self-selected speeds: comfortable, slow, and fast.
  - L-Test: At a comfortable speed, participants were asked to rise from a chair and walk 4 m to a first cone, make a 90° turn to the left around the cone and walk straight to a second cone, turn 180° to the left around the cone and walk straight before making a final 90° turn to the right of the first cone and walking back to sit in the chair.
  - Surface test: From a standing start, participants were asked to walk at a comfortable speed around a circuit by turning around two cones and stepping over a carpeted mat completing the circuit twice.
  - Hallway test: From a standing start, participants were asked to walk at a comfortable speed along a predefined walkway stepping up and down from a step. At the end of the walkway participants completed a sharp 180° turn and walked back along the walkway (again stepping up and down the step) before coming to a stop at the end of the walkway.

Participants were also instructed to resume their typical daily routines, during which multimodal sensor data were continuously collected for 2.5 h. To ensure a diverse capture of real-world gait variability, the execution of specific activities was recommended, including outdoor walking, traversing inclined paths, ascending and descending stairs, and navigating between rooms.

During all assessments, participants were equipped with a multicomponent system of INertial modules, DIstance Sensors and Pressure insoles (INDIP, sampling frequency 100 Hz) [5]. The INDIP system integrated two plantar pressure insoles for a direct measure of foot-to-ground contacts, three IMUs attached to both feet and lower back for activity recognition, turning detection, and displacement estimation, and two time-of-flight infrared distance sensors to detect the alternating movements of the lower extremities [13].

Following the technical verification process, the final dataset for analysis comprised 273 in-lab test trials and 2443 free-living walking bouts (defined as valid gait segments exceeding 10 s in duration and devoid of abrupt turns). The analysis was explicitly restricted to the data derived from the IMU sensor positioned at the lower back.

### 3.3. Measurements and Statistical Analysis

Reference initial contacts (${\mathrm{IC}}_{\mathrm{REF}}$), obtained from the reference systems, were used to evaluate the nine selected step-detection algorithms. Throughout this section, an evaluation unit refers to a single test execution, either an in-laboratory trial or a free-living walking bout. For each evaluation unit and algorithm, the detected ICs were compared with the corresponding ${\mathrm{IC}}_{\mathrm{REF}}$ events to compute the four evaluation metrics described below: detection performance ($F_{1}$-score), initial-contact timing error (${\mathrm{MAE}}_{\mathrm{IC}}$), step-duration error (${\mathrm{MAE}}_{\mathrm{Step}}$), and cadence error ($\%{\mathrm{MRE}}_{\mathrm{cadence}}$).

To enable consistent comparisons across datasets and experimental conditions, evaluation units sharing the same dataset, sensor placement, body side and, where applicable, clinical cohort and recording condition were grouped into an evaluation block. Within each block, the evaluation metrics were aggregated independently for each algorithm across all corresponding evaluation units. Thus, each evaluation block yielded one aggregated value per metric for each of the nine algorithms, allowing the methods to be compared under the same experimental conditions. In total, 37 evaluation blocks were defined: 13 from CorLab, 12 from WearGait-PD (six from the healthy-control cohort and six from the Parkinson’s disease cohort), and 12 from Mobilise-D. For presentation in Table 4, Table 5 and Table 6, the block-level values were further aggregated across body sides, such that separate values for individual sides (e.g., left and right hand or left and right front pocket) are not reported. However, all 37 evaluation blocks were retained as independent units for the subsequent statistical comparisons of the algorithms.

*Event matching and detection performance.* As proposed by Ena et al. [42], a predicted IC was considered a true positive (TP) if it occurred within a ±0.16 s window (0.32 s total) centered on the corresponding ${\mathrm{IC}}_{\mathrm{REF}}$. This tolerance was selected to ensure unique temporal matching between detected and reference events. Specifically, the total tolerance window of 0.32 s corresponds to an inter-step interval at a cadence of 187.5 steps/min (3.125 Hz), such that the matching windows centered on consecutive reference contacts do not overlap at cadences up to this value. Consequently, a detected IC can be associated with at most one reference event. In contrast, a larger tolerance, such as the ±0.25 s used in some studies, would result in a total window of 0.50 s (2 Hz or 120 steps/min), increasing the possibility of overlap with adjacent reference events at higher cadences and potentially inflating detection performance. The ±0.16 s tolerance was therefore used as a conservative criterion for unambiguous temporal matching and is denoted below by τ=0.16 s.

Predicted ICs that did not fall within any ${\mathrm{IC}}_{\mathrm{REF}}$ window were classified as false positives (FP). Conversely, ${\mathrm{IC}}_{\mathrm{REF}}$ events without a corresponding predicted IC were classified as false negatives (FN). Detection performance was quantified for each evaluation unit using the $F_{1}$-score:

(1) $$F_{1}=\frac{\mathrm{TP}}{\mathrm{TP}+0.5(\mathrm{FP}+\mathrm{FN})}.$$

An evaluation unit in which an algorithm detected no TP ICs received an $F_{1}$-score of zero and remained included in the analysis. Within each evaluation block, the $F_{1}$-scores obtained across the corresponding evaluation units were aggregated independently for each algorithm using the arithmetic mean. The resulting block-level $F_{1}$-scores, denoted as $\tilde{F_{1}}$, are reported in Table 4, Table 5 and Table 6 and were used for the subsequent statistical comparison of the algorithms.

*Initial-contact timing error.* For each evaluation unit, the absolute temporal error of TP ICs was quantified in seconds as the median absolute difference between the detected TP IC times and their corresponding ${\mathrm{IC}}_{\mathrm{REF}}$ times:
  (2) $${\mathrm{MAE}}_{\mathrm{IC}}=\underset{i=1,\ldots ,N_{\mathrm{TP}}}{\mathrm{median}}\left|{\mathrm{IC}}_{\mathrm{TP}}^{i}-{\mathrm{IC}}_{\mathrm{REF}}^{i}\right|,$$
  where ${\mathrm{IC}}_{\mathrm{TP}}^{i}$ and ${\mathrm{IC}}_{\mathrm{REF}}^{i}$ denote the times of the *i*th detected TP IC and its corresponding reference initial contact, respectively, and $N_{\mathrm{TP}}$ is the number of TP ICs in the evaluation unit.

The ${\mathrm{MAE}}_{\mathrm{IC}}$ was calculated only for evaluation units containing at least one TP IC. Evaluation units in which an algorithm detected no TP ICs were therefore excluded from the analysis, as the timing error could not be calculated in the absence of TP ICs. Within each evaluation block, the valid evaluation-unit ${\mathrm{MAE}}_{\mathrm{IC}}$ values were aggregated separately for each algorithm using their median. The resulting algorithm-specific block-level median ${\mathrm{MAE}}_{\mathrm{IC}}$ values, denoted as ${\tilde{\mathrm{MAE}}}_{\mathrm{IC}}$, are reported in Table 4, Table 5 and Table 6.

It is important to note that ${\tilde{\mathrm{MAE}}}_{\mathrm{IC}}$ could be based on different numbers of evaluation units for different algorithms. To account for this difference in coverage, $c_{\mathrm{IC}}$ was defined as the proportion of evaluation units within a block for which ${\mathrm{MAE}}_{\mathrm{IC}}$ could be computed. Without a coverage adjustment, an algorithm could obtain a favourable block-level timing error by producing low errors on a small subset of the recordings while failing to detect any valid IC in the remaining units. The maximum admissible timing error, τ, was therefore assigned to the uncovered proportion of the block to account for the potential contribution of undetected ICs:

(3) $${\tilde{\mathrm{MAE}}}_{\mathrm{IC}}^{\mathrm{corr}}=c_{\mathrm{IC}}\;{\tilde{\mathrm{MAE}}}_{\mathrm{IC}}+\left(1-c_{\mathrm{IC}}\right)\;\tau .$$

A predicted IC occurring farther than τ=0.16 s from its corresponding ${\mathrm{IC}}_{\mathrm{REF}}$ is not a TP; therefore, τ is the maximum timing error permitted by the matching criterion. The correction assigns one representative τ-penalised error to each uncovered evaluation unit, irrespective of the number of missed ICs within that unit. It is therefore a coverage-based adjustment rather than an event-level estimate of error. An algorithm with complete coverage ($c_{\mathrm{IC}}=1$) is unaffected, whereas decreasing coverage progressively moves ${\tilde{\mathrm{MAE}}}_{\mathrm{IC}}^{\mathrm{corr}}$ towards τ. Consequently, this correction was applied to reduce the influence of differences in the number of evaluation units across algorithms, and the resulting ${\tilde{\mathrm{MAE}}}_{\mathrm{IC}}^{\mathrm{corr}}$ values were subsequently used for statistical comparison.

*Step-duration error.* A correctly detected step (${\mathrm{Step}}_{\mathrm{TP}}$) was defined by the detection of two consecutive TP ICs, provided that the two corresponding ground-truth ICs constituted a valid step (${\mathrm{Step}}_{\mathrm{REF}}$). For each evaluation unit, the absolute step-duration error was calculated in seconds as:
  (4) $${\mathrm{MAE}}_{\mathrm{Step}}=\underset{j=1,\ldots ,N_{\mathrm{Step}}}{\mathrm{median}}\left|{\mathrm{Step}}_{\mathrm{TP}}^{j}-{\mathrm{Step}}_{\mathrm{REF}}^{j}\right|,$$
  where ${\mathrm{Step}}_{\mathrm{TP}}^{j}$ and ${\mathrm{Step}}_{\mathrm{REF}}^{j}$ denote the *j*th predicted and reference step durations, respectively, and $N_{\mathrm{Step}}$ is the number of correctly detected steps in the evaluation unit.

The ${\mathrm{MAE}}_{\mathrm{Step}}$ was calculated only for evaluation units containing at least one detected step. Evaluation units in which an algorithm detected no steps were therefore excluded from the analysis, as the step-duration error could not be calculated in the absence of steps. Within each evaluation block, the valid evaluation-unit ${\mathrm{MAE}}_{\mathrm{Step}}$ values were aggregated separately for each algorithm using their median. The resulting algorithm-specific block-level median ${\mathrm{MAE}}_{\mathrm{Step}}$ values, denoted as ${\tilde{\mathrm{MAE}}}_{\mathrm{Step}}$, are reported in Table 4, Table 5 and Table 6.

As with IC timing error, ${\tilde{\mathrm{MAE}}}_{\mathrm{Step}}$ could be based on different numbers of evaluation units for different algorithms. Consequently, step-duration coverage, $c_{\mathrm{Step}}$, was defined as the proportion of evaluation units within a block for which ${\mathrm{MAE}}_{\mathrm{Step}}$ could be computed. Since an execution that yields no detected step represents a missed step rather than a timing error, the theoretical maximum penalty is $t_{\max}+2\tau$, where $t_{\max}$ is the maximum plausible step duration. The Mobilise-D pipeline sets the upper limit for stride duration at 3 s, corresponding to $t_{\max}=1.5$ s per step. This penalty was therefore defined as $s_{p}=t_{\max}+2\tau =1.82$ s. The block-level median was adjusted as follows:

(5) $${\tilde{\mathrm{MAE}}}_{\mathrm{Step}}^{\mathrm{corr}}=c_{\mathrm{Step}}\;{\tilde{\mathrm{MAE}}}_{\mathrm{Step}}+\left(1-c_{\mathrm{Step}}\right)\;s_{p}.$$

Consequently, this correction was applied to reduce the influence of differences in the number of evaluation units across algorithms, and the resulting ${\tilde{\mathrm{MAE}}}_{\mathrm{Step}}^{\mathrm{corr}}$ values were subsequently used for statistical comparison.

*Cadence error.* Cadence, expressed in steps/min, was calculated for each evaluation unit as proposed by Micó-Amigo et al. [12]:
  (6) $$\mathrm{Cadence}=2\times \frac{1}{N}\sum_{i=1}^{N}\frac{60}{T_{\mathrm{st}}^{i}},$$
  where a stride was defined as two consecutive steps derived from all detected ICs, *N* is the number of strides, and $T_{\mathrm{st}}^{i}$ is the duration of the *i*th stride. The calculation was applied separately to the predicted and reference IC sequences. Unlike the two temporal-error metrics, predicted cadence was derived from all detected ICs rather than from TP ICs only and therefore did not depend on event matching. A cadence value was defined when at least one valid stride could be formed.

The percentage relative error in cadence was then calculated as:

(7) $$\%{\mathrm{MRE}}_{\mathrm{Cad}}=\frac{\left|{\mathrm{Cadence}}_{\mathrm{Pred}}-{\mathrm{Cadence}}_{\mathrm{REF}}\right|}{{\mathrm{Cadence}}_{\mathrm{REF}}}\times 100,$$

where ${\mathrm{Cadence}}_{\mathrm{Pred}}$ and ${\mathrm{Cadence}}_{\mathrm{REF}}$ denote the predicted cadence and the reference cadence of an evaluation unit, respectively. Within each evaluation block, the evaluation-unit $\%{\mathrm{MRE}}_{\mathrm{Cad}}$ values were aggregated using the median, denoted as ${\tilde{\%\mathrm{MRE}}}_{\mathrm{Cad}}$, and are reported in Table 4, Table 5 and Table 6 and were used for the subsequent statistical comparison of the algorithms.

*Agreement analysis.* The level of agreement between each algorithm’s predictions and the reference measurements of step duration and cadence across evaluation units was assessed within each evaluation block using Bland–Altman analysis [68]. Table 4, Table 5 and Table 6 report the mean difference (bias) and 95% limits of agreement (LoA), defined as the bias ± 1.96 standard deviations of the differences. The Bland–Altman statistics describe agreement with the reference measurements only for those evaluation units for which an algorithm-derived measurement was available.
*Non-parametric comparison across evaluation blocks.* The nine algorithms were compared across the 37 evaluation blocks defined. Because the distributions and scales of the evaluation metrics differed across blocks, and normality and homoscedasticity could not be assumed, a non-parametric approach for comparing multiple algorithms across matched evaluation blocks was adopted following the framework described by Demšar [69]. For each evaluation criterion, the nine algorithms were ranked within each block according to their aggregated block-level value, with rank 1 assigned to the best-performing algorithm.

Differences in the average ranks of the algorithms were assessed using the Friedman test [70], with the Iman–Davenport F-statistic as a refinement [71]. Kendall’s coefficient of concordance, *W*, was additionally reported as an effect-size measure of the consistency of the rankings across evaluation blocks [72]. When the omnibus null hypothesis was rejected, all k(k−1)/2=36 algorithm pairs were compared using the Nemenyi post hoc test [73]. Family-wise error was controlled at α=0.05 using a single critical difference (CD) of 1.97 rank units for k=9 algorithms and N=37 evaluation blocks. The results of the statistical comparison are summarised in Table 7.

### 3.4. Results

This section presents the results by sensor placement, proceeding from the most extensively studied and best-documented location—the pelvis—toward the most distal sites, such as the hands and smartphones. The pelvis (lower back) is the most extensively studied IMU location for gait analysis and the de facto reference site in both clinical and free-living mobility studies; it is also the only placement common to all three datasets analysed in the benchmark comparison (CorLab, WearGait-PD, and Mobilise-D). The comparison is then extended to the remaining sensor placements shared by CorLab and WearGait-PD, and finally to those unique to CorLab. An analysis of the sources of error then follows, based on results aggregated by placement, cohort, and recording environment. The section concludes with a non-parametric statistical comparison of the nine algorithms across all evaluation blocks.

*Sensor placement shared by the three datasets (Pelvis/lower back).* Table 4 shows the three frequency-based methods (FB1, FB2, and FB3) achieved the highest *F*_1_-scores and the lowest timing, step-duration, and cadence errors across CorLab, WearGait-PD, and Mobilise-D. RB1 was also competitive, consistent with its known strength at proximal placements.

In the laboratory tests across the three datasets, FB1, FB2 and FB3 reached $\tilde{F_{1}}$ of 0.86–0.98, with RB1 close behind (0.86–0.95) and step-timing errors of 0.02–0.04 s. This placement additionally enables the only comparison between laboratory and free-living gait, available in Mobilise-D. Under free-living walking, overall performance decreased (FB2 and FB3 reached $\tilde{F_{1}}$ of 0.80–0.90, and AO1 ≤0.43), and cadence estimation degraded sharply for TM1 and RB3 (up to ∼70% for TM1), reflecting the greater variability of unconstrained gait. AO1 was consistently the weakest method; as an adaptive oscillator, it needs a warm-up interval to lock onto the walking rhythm. ML1 returned the lowest $\tilde{F_{1}}$ at this sensor placement in twelve of the fifteen cohort and recording-type combinations (0.18–0.41), and the largest relative cadence errors ${\tilde{\%\mathrm{MRE}}}_{\mathrm{Cad}}$ (20–89%), reflecting a low detection rate rather than imprecise timing: its uncorrected ${\tilde{\mathrm{MAE}}}_{\mathrm{IC}}$ of 0.02–0.06 s was comparable to that of FB1, FB2 and FB3.

The Bland–Altman analysis showed that FB3 combined a near-zero bias with the narrowest step-duration limits (e.g., −0.024 to +0.028 s in WearGait-PD), with FB1 and FB2 only slightly wider, and the frequency methods’ cadence limits were commonly tighter in the laboratory than in free-living walking. TM1 and RB3 developed large positive cadence biases under free-living conditions (+44.1 and +43.4 steps/min, respectively), consistent with their degraded cadence errors and a tendency toward cadence overestimation.

**Table 4:** Step-detection performance and Bland–Altman (BA) agreement at the **Pelvis/lower-back** placement, across all datasets and cohorts. Rows are grouped into metric blocks (Score column), reported top to bottom as: $\tilde{F_{1}}$, ${\tilde{\mathrm{MAE}}}_{\mathrm{IC}}$, ${\tilde{\mathrm{MAE}}}_{\mathrm{Step}}$, ${\mathrm{MAE}}_{\mathrm{Step}}^{\mathrm{BA}}$, ${\tilde{\%\mathrm{MRE}}}_{\mathrm{Cad}}$, and ${\mathrm{Cad}}^{\mathrm{BA}}$. $\tilde{F_{1}}$ is given as mean ± SD and the error metrics (${\tilde{\mathrm{MAE}}}_{\mathrm{IC}}$, ${\tilde{\mathrm{MAE}}}_{\mathrm{Step}}$ and ${\tilde{\%\mathrm{MRE}}}_{\mathrm{Cad}}$) as median [IQR]. The two BA-based blocks (${\mathrm{MAE}}_{\mathrm{Step}}^{\mathrm{BA}}$ and ${\mathrm{Cad}}^{\mathrm{BA}}$) report agreement between predictions and ground truth as bias [LL,UL], where the bias is mean(Pred − GT) and LL, UL denote the lower and upper 95% limits of agreement. Best method(s) per column in **bold** (ties all shown); the BA blocks are not bolded. Column headers give cohort and condition: L = structured laboratory tests, FL = free-living walking bouts. *n* (under each column header) is the number of test executions.

Score	Method	CorLab	WearGait-PD		Mobilise-D											
		HC	HC	PD	HC		PD		MS		PFF		COPD		CHF	
		L	L	L	L	FL	L	FL	L	FL	L	FL	L	FL	L	FL
		*n* = 982	*n* = 415	*n* = 490	*n* = 44	*n* = 690	*n* = 40	*n* = 301	*n* = 65	*n* = 293	*n* = 47	*n* = 299	*n* = 64	*n* = 507	*n* = 13	*n* = 353
$\tilde{F_{1}}$	AO1	0.30 ±0.17	0.43 ±0.21	0.44 ±0.20	0.31 ±0.18	0.41 ±0.23	0.25 ±0.18	0.43 ±0.25	0.27 ±0.17	0.38 ±0.21	0.38 ±0.16	0.38 ±0.19	0.37 ±0.20	0.37 ±0.22	0.26 ±0.18	0.37 ±0.24
	TM1	0.67 ±0.14	0.81 ±0.14	0.79 ±0.14	0.73 ±0.14	0.64 ±0.18	0.70 ±0.16	0.66 ±0.18	0.70 ±0.16	0.66 ±0.16	0.68 ±0.12	0.62 ±0.14	0.77 ±0.13	0.60 ±0.18	0.76 ±0.23	0.69 ±0.18
	RB1	0.90 ±0.09	0.87 ±0.14	0.88 ±0.14	0.86 ±0.15	0.85 ±0.18	0.95 ±0.06	**0.88** ±0.17	0.90 ±0.11	0.84 ±0.20	0.89 ±0.11	**0.86** ±0.16	0.95 ±0.06	0.83 ±0.20	**0.95** ±0.05	0.86 ±0.17
	RB2	0.89 ±0.16	0.83 ±0.17	0.84 ±0.18	0.74 ±0.20	0.49 ±0.34	0.66 ±0.27	0.47 ±0.32	0.59 ±0.30	0.46 ±0.29	0.67 ±0.23	0.57 ±0.26	0.65 ±0.26	0.38 ±0.28	0.58 ±0.36	0.71 ±0.27
	RB3	0.61 ±0.14	0.55 ±0.17	0.57 ±0.17	0.55 ±0.19	0.54 ±0.17	0.51 ±0.18	0.52 ±0.16	0.52 ±0.15	0.51 ±0.16	0.59 ±0.15	0.58 ±0.15	0.52 ±0.18	0.52 ±0.17	0.56 ±0.17	0.55 ±0.15
	FB1	**0.98** ±0.04	0.94 ±0.13	0.92 ±0.16	0.93 ±0.09	0.80 ±0.23	0.95 ±0.06	0.77 ±0.24	0.88 ±0.18	0.81 ±0.23	0.86 ±0.15	0.81 ±0.19	0.90 ±0.11	0.66 ±0.28	0.94 ±0.10	0.85 ±0.20
	FB2	**0.98** ±0.04	**0.98** ±0.05	0.97 ±0.06	**0.95** ±0.10	**0.88** ±0.18	**0.97** ±0.03	**0.88** ±0.18	**0.92** ±0.14	**0.87** ±0.18	**0.93** ±0.11	**0.86** ±0.16	**0.98** ±0.05	**0.84** ±0.20	0.94 ±0.11	**0.90** ±0.15
	FB3	**0.98** ±0.05	**0.98** ±0.06	**0.98** ±0.06	**0.95** ±0.10	0.85 ±0.19	0.96 ±0.07	0.86 ±0.20	0.90 ±0.17	**0.87** ±0.17	0.91 ±0.13	0.83 ±0.19	0.97 ±0.03	0.80 ±0.22	0.92 ±0.11	0.89 ±0.16
	ML1	0.21 ±0.20	0.35 ±0.25	0.41 ±0.26	0.31 ±0.26	0.20 ±0.19	0.27 ±0.25	0.26 ±0.20	0.27 ±0.23	0.22 ±0.20	0.18 ±0.15	0.25 ±0.17	0.28 ±0.23	0.21 ±0.23	0.18 ±0.16	0.35 ±0.22
${\tilde{\mathrm{MAE}}}_{\mathrm{IC}}$ (s)	AO1	0.14 [0.12–0.16]	0.12 [0.09–0.14]	0.12 [0.10–0.14]	0.12 [0.10–0.14]	0.10 [0.08–0.12]	0.12 [0.08–0.14]	0.10 [0.08–0.14]	0.11 [0.08–0.14]	0.10 [0.08–0.12]	0.09 [0.08–0.12]	0.10 [0.07–0.12]	0.12 [0.10–0.14]	0.09 [0.06–0.12]	0.10 [0.08–0.13]	0.12 [0.08–0.14]
	TM1	**0.04** [0.04–0.06]	0.08 [0.06–0.10]	0.08 [0.06–0.10]	0.07 [0.05–0.08]	0.09 [0.07–0.10]	0.08 [0.06–0.10]	0.10 [0.08–0.12]	0.08 [0.06–0.10]	0.10 [0.08–0.12]	0.08 [0.06–0.09]	0.08 [0.06–0.10]	0.08 [0.06–0.10]	0.10 [0.08–0.12]	0.06 [0.06–0.10]	0.08 [0.06–0.10]
	RB1	0.06 [0.04–0.08]	0.05 [0.04–0.06]	0.05 [0.04–0.06]	0.04 [0.03–0.06]	**0.04** [0.04–0.06]	0.04 [0.02–0.06]	**0.04** [0.03–0.06]	0.05 [0.04–0.07]	**0.04** [0.02–0.06]	**0.04** [0.04–0.06]	0.04 [0.03–0.06]	0.04 [0.02–0.06]	**0.04** [0.03–0.06]	0.04 [0.02–0.04]	0.06 [0.04–0.08]
	RB2	0.10 [0.08–0.12]	0.10 [0.08–0.12]	0.10 [0.08–0.12]	0.12 [0.10–0.14]	0.12 [0.10–0.14]	0.13 [0.11–0.14]	0.13 [0.11–0.15]	0.12 [0.09–0.14]	0.14 [0.10–0.14]	0.12 [0.11–0.14]	0.13 [0.12–0.14]	0.13 [0.12–0.14]	0.14 [0.11–0.15]	0.12 [0.11–0.16]	0.10 [0.08–0.13]
	RB3	0.08 [0.06–0.10]	0.08 [0.06–0.12]	0.09 [0.06–0.11]	0.10 [0.08–0.12]	0.10 [0.08–0.12]	0.09 [0.06–0.10]	0.10 [0.08–0.12]	0.08 [0.06–0.10]	0.10 [0.08–0.12]	0.10 [0.08–0.11]	0.08 [0.07–0.10]	0.10 [0.08–0.12]	0.10 [0.07–0.12]	0.11 [0.10–0.12]	0.10 [0.06–0.12]
	FB1	0.06 [0.04–0.08]	0.08 [0.07–0.10]	0.08 [0.06–0.10]	0.08 [0.06–0.08]	0.10 [0.08–0.12]	0.10 [0.07–0.10]	0.10 [0.08–0.12]	0.08 [0.06–0.10]	0.10 [0.08–0.12]	0.08 [0.07–0.11]	0.08 [0.06–0.10]	0.10 [0.07–0.12]	0.11 [0.08–0.13]	0.08 [0.08–0.10]	0.08 [0.07–0.10]
	FB2	**0.04** [0.02–0.06]	**0.02** [0.02–0.04]	**0.02** [0.02–0.04]	**0.02** [0.02–0.04]	**0.04** [0.02–0.05]	**0.02** [0.02–0.04]	**0.04** [0.02–0.06]	**0.04** [0.02–0.06]	**0.04** [0.02–0.06]	**0.04** [0.02–0.04]	0.04 [0.03–0.06]	0.04 [0.02–0.04]	0.05 [0.04–0.07]	0.04 [0.02–0.04]	0.03 [0.02–0.04]
	FB3	**0.04** [0.02–0.06]	**0.02** [0.02–0.04]	**0.02** [0.02–0.04]	**0.02** [0.02–0.03]	**0.04** [0.02–0.06]	**0.02** [0.02–0.04]	**0.04** [0.02–0.06]	**0.04** [0.02–0.05]	**0.04** [0.02–0.06]	**0.04** [0.02–0.04]	0.04 [0.02–0.06]	**0.03** [0.02–0.04]	0.06 [0.04–0.08]	**0.02** [0.02–0.04]	**0.02** [0.02–0.04]
	ML1	**0.04** [0.02–0.08]	0.04 [0.02–0.06]	0.04 [0.02–0.06]	0.04 [0.02–0.06]	**0.04** [0.02–0.06]	0.04 [0.02–0.04]	**0.04** [0.02–0.06]	**0.04** [0.02–0.06]	**0.04** [0.03–0.06]	**0.04** [0.02–0.04]	**0.02** [0.02–0.04]	0.04 [0.02–0.05]	0.05 [0.04–0.08]	0.06 [0.06–0.09]	0.04 [0.02–0.04]
${\tilde{\mathrm{MAE}}}_{\mathrm{Step}}$ (s)	AO1	**0.02** [0.02–0.05]	**0.02** [0.02–0.04]	**0.02** [0.02–0.04]	0.03 [0.02–0.06]	0.05 [0.02–0.10]	0.07 [0.04–0.10]	0.04 [0.02–0.08]	0.05 [0.04–0.08]	0.06 [0.04–0.10]	0.06 [0.04–0.09]	0.07 [0.04–0.11]	0.04 [0.02–0.06]	0.08 [0.04–0.12]	**0.02** [0.02–0.07]	0.04 [0.02–0.08]
	TM1	**0.02** [0.02–0.04]	**0.02** [0.02–0.04]	**0.02** [0.02–0.06]	0.03 [0.02–0.04]	0.04 [0.02–0.11]	0.04 [0.02–0.11]	0.05 [0.04–0.09]	0.06 [0.04–0.11]	0.08 [0.04–0.12]	0.05 [0.04–0.08]	0.06 [0.04–0.12]	0.04 [0.02–0.06]	0.08 [0.04–0.14]	**0.02** [0.02–0.03]	0.03 [0.02–0.08]
	RB1	0.04 [0.04–0.06]	0.06 [0.04–0.08]	0.04 [0.04–0.07]	0.06 [0.04–0.08]	0.05 [0.03–0.07]	0.04 [0.03–0.06]	0.06 [0.04–0.08]	0.06 [0.04–0.09]	0.05 [0.04–0.08]	0.04 [0.03–0.08]	0.04 [0.02–0.06]	0.04 [0.03–0.07]	0.06 [0.04–0.08]	0.04 [0.02–0.06]	0.06 [0.04–0.08]
	RB2	0.03 [0.02–0.04]	0.03 [0.02–0.04]	**0.02** [0.02–0.04]	0.04 [0.02–0.05]	**0.02** [0.02–0.04]	**0.02** [0.02–0.02]	**0.02** [0.02–0.04]	**0.04** [0.02–0.08]	**0.02** [0.02–0.05]	**0.02** [0.02–0.04]	**0.02** [0.02–0.03]	**0.02** [0.02–0.04]	**0.03** [0.02–0.04]	**0.02** [0.01–0.02]	**0.02** [0.02–0.04]
	RB3	0.06 [0.04–0.09]	0.06 [0.03–0.10]	0.06 [0.04–0.10]	0.06 [0.04–0.11]	0.09 [0.06–0.14]	0.08 [0.07–0.17]	0.10 [0.06–0.14]	0.10 [0.06–0.16]	0.10 [0.06–0.14]	0.09 [0.06–0.14]	0.09 [0.06–0.12]	0.08 [0.04–0.17]	0.10 [0.06–0.14]	0.07 [0.06–0.09]	0.08 [0.04–0.12]
	FB1	**0.02** [0.02–0.02]	**0.02** [0.02–0.02]	**0.02** [0.02–0.02]	**0.02** [0.02–0.04]	**0.02** [0.02–0.04]	**0.02** [0.02–0.04]	**0.02** [0.02–0.04]	**0.04** [0.02–0.07]	**0.02** [0.02–0.04]	0.04 [0.02–0.06]	0.04 [0.02–0.06]	**0.02** [0.02–0.04]	0.04 [0.02–0.06]	**0.02** [0.02–0.02]	**0.02** [0.02–0.04]
	FB2	**0.02** [0.02–0.02]	**0.02** [0.02–0.02]	**0.02** [0.02–0.02]	**0.02** [0.02–0.04]	0.03 [0.02–0.05]	**0.02** [0.02–0.04]	0.04 [0.02–0.06]	**0.04** [0.02–0.06]	0.04 [0.02–0.05]	0.04 [0.02–0.04]	0.04 [0.02–0.06]	**0.02** [0.02–0.04]	0.04 [0.02–0.08]	**0.02** [0.02–0.03]	**0.02** [0.02–0.04]
	FB3	**0.02** [0.02–0.02]	**0.02** [0.02–0.02]	**0.02** [0.02–0.02]	**0.02** [0.02–0.03]	0.04 [0.02–0.06]	**0.02** [0.02–0.04]	0.04 [0.02–0.06]	**0.04** [0.02–0.06]	0.04 [0.02–0.06]	0.04 [0.02–0.04]	0.04 [0.02–0.06]	0.03 [0.02–0.04]	0.05 [0.04–0.08]	**0.02** [0.01–0.02]	**0.02** [0.02–0.06]
	ML1	0.04 [0.02–0.09]	**0.02** [0.02–0.04]	**0.02** [0.02–0.04]	0.04 [0.02–0.08]	0.03 [0.02–0.06]	0.06 [0.02–0.06]	0.04 [0.03–0.08]	0.06 [0.04–0.11]	0.04 [0.02–0.08]	**0.02** [0.02–0.10]	0.04 [0.02–0.07]	0.04 [0.01–0.06]	0.04 [0.02–0.07]	**0.02** [0.02–0.07]	0.04 [0.02–0.08]
${\mathrm{MAE}}_{\mathrm{Step}}^{\mathrm{BA}}$ (s)	AO1	−0.007 [−0.11, 0.09]	−0.006 [−0.07, 0.06]	−0.005 [−0.09, 0.08]	−0.001 [−0.08, 0.08]	0.001 [−0.14, 0.14]	−0.014 [−0.21, 0.18]	−0.004 [−0.13, 0.12]	0.001 [−0.13, 0.13]	−0.004 [−0.14, 0.14]	−0.007 [−0.12, 0.11]	−0.009 [−0.17, 0.15]	0.007 [−0.10, 0.12]	−0.004 [−0.17, 0.16]	0.025 [−0.06, 0.11]	−0.007 [−0.15, 0.14]
	TM1	−0.001 [−0.05, 0.05]	0.004 [−0.05, 0.06]	0.006 [−0.06, 0.07]	−0.016 [−0.10, 0.07]	−0.002 [−0.13, 0.13]	0.013 [−0.10, 0.12]	0.002 [−0.12, 0.12]	−0.003 [−0.10, 0.10]	0.007 [−0.11, 0.12]	0.004 [−0.10, 0.11]	0.007 [−0.11, 0.12]	−0.005 [−0.09, 0.08]	−0.003 [−0.16, 0.15]	−0.005 [−0.07, 0.06]	0.001 [−0.10, 0.11]
	RB1	0.023 [−0.03, 0.08]	0.033 [−0.06, 0.12]	0.033 [−0.04, 0.11]	0.020 [−0.08, 0.12]	0.013 [−0.07, 0.09]	0.020 [−0.04, 0.08]	0.007 [−0.07, 0.09]	0.013 [−0.08, 0.11]	0.011 [−0.08, 0.10]	0.016 [−0.04, 0.08]	0.021 [−0.07, 0.11]	0.010 [−0.06, 0.08]	0.004 [−0.09, 0.10]	0.008 [−0.04, 0.06]	0.016 [−0.07, 0.10]
	RB2	0.016 [−0.03, 0.06]	0.024 [−0.05, 0.09]	0.027 [−0.05, 0.10]	0.006 [−0.07, 0.08]	0.007 [−0.06, 0.08]	0.005 [−0.04, 0.05]	0.004 [−0.04, 0.04]	−0.007 [−0.11, 0.09]	−0.001 [−0.07, 0.07]	0.005 [−0.06, 0.07]	0.007 [−0.09, 0.11]	−0.003 [−0.05, 0.04]	0.006 [−0.09, 0.10]	0.008 [−0.04, 0.06]	0.001 [−0.06, 0.06]
	RB3	0.001 [−0.08, 0.08]	0.004 [−0.10, 0.11]	0.007 [−0.11, 0.13]	−0.001 [−0.14, 0.14]	−0.001 [−0.16, 0.16]	−0.004 [−0.22, 0.21]	0.004 [−0.17, 0.17]	0.008 [−0.17, 0.18]	0.004 [−0.17, 0.18]	0.031 [−0.11, 0.17]	0.010 [−0.11, 0.13]	0.018 [−0.14, 0.18]	0.004 [−0.18, 0.18]	−0.011 [−0.15, 0.13]	0.010 [−0.14, 0.16]
	FB1	0.001 [−0.02, 0.03]	0.006 [−0.02, 0.04]	0.006 [−0.03, 0.04]	−0.005 [−0.06, 0.05]	−0.001 [−0.06, 0.06]	0.002 [−0.04, 0.05]	0.000 [−0.05, 0.05]	−0.005 [−0.08, 0.07]	−0.000 [−0.05, 0.05]	0.003 [−0.05, 0.06]	0.006 [−0.07, 0.08]	−0.002 [−0.04, 0.04]	−0.004 [−0.09, 0.08]	0.012 [−0.02, 0.05]	0.001 [−0.05, 0.06]
	FB2	−0.001 [−0.03, 0.02]	0.006 [−0.02, 0.04]	0.007 [−0.02, 0.04]	−0.001 [−0.04, 0.04]	−0.000 [−0.07, 0.07]	−0.002 [−0.05, 0.04]	0.000 [−0.07, 0.07]	−0.011 [−0.09, 0.06]	−0.000 [−0.06, 0.06]	0.007 [−0.05, 0.06]	0.005 [−0.06, 0.07]	−0.007 [−0.05, 0.04]	−0.005 [−0.10, 0.09]	0.010 [−0.02, 0.04]	−0.002 [−0.06, 0.05]
	FB3	−0.004 [−0.03, 0.02]	0.002 [−0.03, 0.03]	0.002 [−0.02, 0.03]	−0.001 [−0.04, 0.04]	−0.007 [−0.09, 0.07]	−0.007 [−0.07, 0.06]	−0.007 [−0.08, 0.07]	−0.016 [−0.08, 0.05]	−0.006 [−0.08, 0.07]	−0.000 [−0.04, 0.04]	−0.005 [−0.08, 0.07]	−0.008 [−0.05, 0.04]	−0.007 [−0.10, 0.08]	0.001 [−0.03, 0.03]	−0.009 [−0.08, 0.06]
	ML1	−0.002 [−0.14, 0.14]	0.003 [−0.08, 0.09]	−0.001 [−0.10, 0.09]	0.045 [−0.11, 0.20]	−0.003 [−0.10, 0.09]	−0.033 [−0.19, 0.12]	0.001 [−0.12, 0.12]	−0.004 [−0.16, 0.15]	−0.010 [−0.12, 0.10]	−0.016 [−0.14, 0.11]	0.000 [−0.10, 0.10]	0.012 [−0.11, 0.13]	−0.001 [−0.10, 0.10]	0.053 [−0.06, 0.17]	−0.002 [−0.10, 0.09]
${\tilde{\%\mathrm{MRE}}}_{\mathrm{Cad}}$	AO1	5.0 [2.1–13.3]	2.9 [0.9–6.8]	3.0 [1.2–6.9]	4.7 [1.9–7.8]	11.8 [3.0–37.7]	7.0 [3.1–15.5]	7.5 [1.3–33.9]	6.7 [3.3–18.1]	14.4 [3.7–45.7]	12.9 [4.2–54.5]	21.2 [8.0–62.2]	3.9 [1.9–7.4]	14.6 [5.2–38.0]	12.3 [2.9–14.3]	7.2 [2.1–23.8]
	TM1	9.6 [2.1–25.2]	12.6 [6.4–23.9]	13.1 [5.9–23.5]	12.2 [3.1–24.5]	49.0 [15.5–89.7]	28.2 [4.8–50.7]	29.8 [8.9–80.3]	37.0 [7.4–63.6]	54.8 [20.8–94.3]	59.1 [28.5–107.6]	70.4 [37.5–107.4]	10.5 [3.1–24.0]	59.3 [31.5–92.5]	12.5 [5.9–23.1]	20.8 [6.0–67.0]
	RB1	8.2 [4.3–14.6]	6.6 [2.2–19.8]	5.2 [1.5–18.5]	5.2 [1.7–16.2]	4.8 [1.4–13.7]	2.0 [1.3–4.5]	3.5 [1.2–13.2]	3.3 [1.4–8.5]	4.1 [0.8–15.8]	3.3 [0.7–13.0]	4.4 [1.2–13.3]	1.6 [0.6–4.7]	4.6 [1.3–12.0]	1.9 [0.8–10.0]	3.4 [0.4–14.9]
	RB2	6.7 [4.0–12.0]	4.8 [1.8–17.2]	4.2 [1.5–16.1]	5.1 [1.4–20.8]	5.0 [1.1–12.7]	2.1 [1.4–4.1]	3.1 [0.7–12.4]	3.1 [1.6–7.3]	3.0 [0.8–13.4]	5.1 [1.3–9.1]	4.7 [1.0–13.5]	1.6 [0.9–3.7]	4.3 [1.3–12.1]	2.6 [1.1–10.8]	2.6 [0.3–12.7]
	RB3	19.6 [9.4–31.6]	11.9 [4.1–26.7]	16.2 [5.9–29.8]	21.0 [5.3–43.2]	48.6 [23.0–81.7]	33.3 [21.6–55.9]	38.6 [16.1–76.2]	41.1 [22.9–62.8]	49.4 [33.2–81.0]	44.6 [24.9–80.3]	65.2 [40.5–96.2]	29.5 [10.1–48.4]	63.2 [35.7–92.3]	24.2 [17.5–55.9]	38.3 [19.9–68.9]
	FB1	2.6 [2.0–5.0]	0.8 [0.4–1.6]	0.9 [0.4–2.2]	**1.1** [0.4–3.0]	**2.0** [0.4–7.6]	1.9 [1.0–3.4]	**1.3** [0.2–5.6]	1.8 [0.7–3.3]	**1.6** [0.4–7.1]	1.7 [0.6–5.5]	**3.6** [0.8–12.7]	**1.0** [0.4–2.1]	**3.2** [0.9–9.7]	1.1 [0.3–3.5]	**1.1** [0.2–5.8]
	FB2	2.4 [2.0–4.8]	0.9 [0.4–1.7]	1.1 [0.4–2.6]	1.2 [0.4–2.2]	2.3 [0.4–10.0]	1.7 [0.8–3.2]	1.6 [0.2–10.0]	**1.3** [0.4–4.6]	2.0 [0.5–8.9]	1.8 [0.6–8.4]	5.7 [1.5–14.4]	**1.1** [0.5–1.8]	3.4 [1.3–13.3]	**1.0** [0.7–4.8]	1.2 [0.2–7.2]
	FB3	**2.2** [1.8–4.7]	**0.7** [0.3–1.5]	**0.8** [0.3–1.9]	2.2 [1.0–7.8]	3.4 [0.6–15.7]	**1.2** [0.4–3.0]	1.5 [0.2–13.9]	1.6 [0.6–5.1]	2.3 [0.5–8.9]	**1.3** [0.4–13.4]	5.1 [1.3–14.6]	1.2 [0.7–2.6]	5.4 [1.2–19.9]	1.7 [0.8–11.1]	1.8 [0.2–12.6]
	ML1	88.6 [76.2–95.7]	44.8 [22.9–54.0]	42.8 [24.2–50.1]	37.4 [29.8–54.8]	66.4 [48.9–79.4]	28.6 [7.9–50.0]	66.5 [49.5–79.2]	32.2 [17.7–48.3]	67.3 [49.3–78.2]	58.4 [28.5–74.5]	66.6 [49.3–76.1]	25.0 [8.0–49.7]	49.8 [33.2–65.8]	20.3 [10.7–45.1]	60.7 [41.0–73.5]
${\mathrm{Cad}}^{\mathrm{BA}}$ (spm)	AO1	−4.3 [−23, 15]	0.0 [−26, 26]	−1.4 [−21, 18]	0.4 [−14, 15]	10.7 [−51, 72]	5.7 [−34, 46]	11.6 [−50, 73]	2.3 [−45, 49]	10.3 [−56, 77]	13.7 [−56, 83]	13.7 [−59, 86]	0.9 [−13, 15]	11.3 [−50, 72]	4.0 [−42, 50]	9.1 [−45, 63]
	TM1	10.6 [−20, 42]	18.9 [−18, 56]	17.8 [−16, 52]	13.2 [−18, 44]	44.1 [−18, 106]	26.8 [−20, 74]	37.5 [−26, 101]	35.8 [−28, 99]	46.4 [−14, 107]	50.3 [−7, 108]	55.1 [6, 104]	13.5 [−21, 48]	49.5 [−3, 102]	22.4 [−31, 76]	31.0 [−25, 87]
	RB1	−8.0 [−25, 9]	−15.3 [−55, 24]	−14.1 [−55, 27]	−11.2 [−50, 27]	−3.8 [−33, 25]	0.3 [−20, 21]	−3.2 [−29, 22]	−2.6 [−22, 17]	−4.1 [−35, 26]	−4.4 [−24, 16]	−2.8 [−31, 26]	−2.5 [−20, 15]	−0.6 [−25, 24]	−2.8 [−15, 10]	−3.6 [−33, 26]
	RB2	−6.7 [−21, 8]	−14.2 [−51, 23]	−13.8 [−55, 27]	−12.7 [−51, 25]	−2.3 [−31, 26]	−1.4 [−10, 8]	−1.8 [−26, 22]	−0.9 [−19, 17]	−1.8 [−29, 26]	−1.2 [−18, 15]	−1.4 [−27, 25]	−2.8 [−16, 10]	0.8 [−23, 25]	−2.1 [−18, 13]	−2.3 [−29, 24]
	RB3	15.9 [−9, 41]	16.8 [−16, 50]	18.6 [−16, 53]	23.9 [−14, 61]	43.4 [−11, 98]	36.4 [−9, 82]	39.9 [−17, 97]	38.2 [−6, 82]	45.8 [1, 90]	44.0 [−2, 90]	51.4 [10, 93]	26.9 [−15, 69]	51.9 [−1, 105]	37.7 [−17, 93]	38.6 [−11, 88]
	FB1	−2.3 [−7, 2]	−1.0 [−7, 5]	−1.9 [−15, 11]	−1.4 [−7, 4]	−1.0 [−20, 18]	−0.1 [−9, 8]	0.7 [−18, 19]	−1.6 [−12, 8]	−0.0 [−16, 16]	−1.0 [−18, 16]	−2.7 [−28, 23]	−0.3 [−6, 5]	−0.6 [−23, 22]	5.3 [−23, 34]	0.4 [−17, 18]
	FB2	−1.7 [−7, 3]	−0.9 [−10, 8]	−1.6 [−16, 13]	−0.9 [−7, 5]	4.2 [−19, 28]	1.0 [−8, 10]	5.7 [−21, 32]	0.8 [−20, 22]	4.1 [−20, 28]	4.2 [−21, 30]	4.6 [−21, 30]	−0.2 [−5, 4]	4.6 [−24, 33]	7.6 [−35, 50]	4.0 [−17, 25]
	FB3	0.3 [−17, 17]	−0.6 [−9, 8]	−1.0 [−15, 13]	2.7 [−12, 17]	7.2 [−26, 40]	1.4 [−10, 13]	7.3 [−24, 38]	3.3 [−24, 31]	6.1 [−24, 36]	8.4 [−26, 43]	6.8 [−24, 38]	1.9 [−9, 13]	8.1 [−25, 41]	11.0 [−30, 52]	5.4 [−20, 31]
	ML1	−65.9 [−121, −11]	−41.5 [−94, 11]	−38.6 [−91, 14]	−38.2 [−80, 4]	−56.0 [−121, 9]	−10.6 [−93, 72]	−54.0 [−131, 23]	−27.4 [−73, 18]	−53.9 [−105, −2]	−19.5 [−141, 101]	−46.9 [−108, 15]	−23.6 [−78, 31]	−39.1 [−97, 19]	−29.8 [−94, 35]	−52.2 [−100, −4]

**Table 5:** Sensor placements shared by the CorLab and WearGait-PD datasets (Mid-Upper Leg, Forearm, Head). There is one column group per metric, each split into CorLab (HC) and WearGait-PD (HC and PD). $\tilde{F_{1}}$ is given as mean ± SD and the error metrics (${\tilde{\mathrm{MAE}}}_{\mathrm{IC}}$, ${\tilde{\mathrm{MAE}}}_{\mathrm{Step}}$, ${\tilde{\%\mathrm{MRE}}}_{\mathrm{Cad}}$) as median [IQR]. The two BA-based column groups (${\mathrm{MAE}}_{\mathrm{Step}}^{\mathrm{BA}}$, ${\mathrm{Cad}}^{\mathrm{BA}}$) report agreement between predictions and ground truth as bias [LL, UL], where the bias is mean(Pred − GT) and LL, UL denote the lower and upper 95% limits of agreement. Best method(s) per column in **bold** (ties all shown); the BA groups are not bolded. *n* denotes the number of test executions for CorLab/WearGait-PD HC/WearGait-PD PD, respectively.

Placement	Method	$\tilde{F_{1}}$			${\tilde{\mathrm{MAE}}}_{\mathrm{IC}}$ (s)			${\tilde{\mathrm{MAE}}}_{\mathrm{Step}}$ (s)			${\mathrm{MAE}}_{\mathrm{Step}}^{\mathrm{BA}}$ (s)			${\tilde{\%\mathrm{MRE}}}_{\mathrm{Cad}}$			${\mathrm{Cad}}^{\mathrm{BA}}$ (spm)		
		CorLab	WearGait-PD		CorLab	WearGait-PD		CorLab	WearGait-PD		CorLab	WearGait-PD		CorLab	WearGait-PD		CorLab	WearGait-PD	
		HC	HC	PD	HC	HC	PD	HC	HC	PD	HC	HC	PD	HC	HC	PD	HC	HC	PD
Mid-Upper Leg (*n* = 1964/830/980)	AO1	0.45 ±0.12	0.51 ±0.15	0.51 ±0.16	0.10 [0.08–0.12]	0.12 [0.10–0.13]	0.11 [0.09–0.12]	0.14 [0.08–0.18]	0.07 [0.04–0.15]	0.08 [0.04–0.14]	0.004 [−0.39, 0.40]	0.005 [−0.53, 0.54]	0.011 [−0.62, 0.65]	20.0 [9.4–42.0]	7.2 [2.6–19.8]	7.3 [2.7–16.7]	9.0 [−35, 54]	10.2 [−31, 52]	8.0 [−30, 46]
	TM1	0.71 ±0.08	0.71 ±0.11	0.72 ±0.12	0.04 [0.04–0.06]	0.06 [0.04–0.08]	0.06 [0.04–0.08]	0.08 [0.06–0.11]	0.10 [0.06–0.12]	0.10 [0.06–0.12]	0.006 [−0.15, 0.16]	0.012 [−0.27, 0.29]	0.014 [−0.27, 0.30]	43.8 [33.3–51.0]	30.6 [12.7–47.5]	32.1 [13.2–47.4]	31.6 [2, 61]	32.9 [−7, 73]	32.7 [−7, 72]
	RB1	0.58 ±0.22	0.50 ±0.25	0.50 ±0.26	0.06 [0.04–0.09]	0.10 [0.08–0.12]	0.10 [0.06–0.13]	0.14 [0.10–0.18]	0.18 [0.14–0.22]	0.17 [0.12–0.22]	0.038 [−0.37, 0.45]	0.035 [−0.46, 0.53]	0.049 [−0.66, 0.76]	35.3 [18.6–47.9]	43.4 [25.8–49.9]	41.5 [22.7–49.4]	−26.6 [−64, 11]	−41.6 [−87, 4]	−39.1 [−88, 9]
	RB2	0.75 ±0.13	0.65 ±0.12	0.69 ±0.13	0.05 [0.02–0.08]	0.06 [0.03–0.08]	0.06 [0.04–0.08]	0.08 [0.06–0.10]	0.06 [0.04–0.08]	0.06 [0.04–0.08]	0.002 [−0.36, 0.36]	0.008 [−0.60, 0.62]	0.007 [−0.60, 0.61]	27.6 [11.8–43.5]	41.1 [21.0–49.6]	39.1 [16.4–49.2]	−22.6 [−59, 14]	−39.9 [−85, 5]	−37.0 [−84, 10]
	RB3	0.65 ±0.10	0.71 ±0.12	0.72 ±0.12	0.08 [0.06–0.09]	0.08 [0.06–0.10]	0.08 [0.06–0.10]	0.12 [0.08–0.14]	0.10 [0.08–0.14]	0.10 [0.08–0.14]	0.008 [−0.33, 0.35]	0.016 [−0.40, 0.43]	0.021 [−0.30, 0.34]	29.2 [16.0–42.9]	21.1 [9.3–38.9]	23.0 [10.9–40.3]	22.1 [−4, 49]	24.9 [−13, 62]	25.7 [−11, 62]
	FB1	0.96 ±0.07	0.92 ±0.13	0.90 ±0.14	0.08 [0.06–0.08]	0.10 [0.08–0.11]	0.10 [0.08–0.12]	0.04 [0.02–0.06]	**0.02** [0.02–0.04]	**0.02** [0.02–0.04]	0.001 [−0.09, 0.09]	0.005 [−0.14, 0.15]	0.005 [−0.19, 0.20]	2.3 [2.0–4.8]	1.0 [0.3–2.6]	0.9 [0.4–2.5]	−2.0 [−7, 3]	−1.5 [−21, 18]	−2.0 [−24, 20]
	FB2	**0.98** ±0.03	**0.97** ±0.07	**0.97** ±0.06	**0.02** [0.02–0.04]	**0.02** [0.02–0.04]	**0.02** [0.02–0.04]	**0.02** [0.02–0.04]	**0.02** [0.02–0.04]	0.03 [0.02–0.04]	−0.003 [−0.04, 0.04]	0.006 [−0.07, 0.08]	0.006 [−0.20, 0.21]	**2.2** [0.0–4.5]	0.9 [0.4–2.5]	1.0 [0.4–2.6]	−1.0 [−6, 4]	−1.4 [−21, 18]	−2.0 [−27, 23]
	FB3	**0.98** ±0.04	0.96 ±0.08	0.96 ±0.06	0.04 [0.04–0.06]	0.06 [0.06–0.08]	0.08 [0.06–0.09]	**0.02** [0.02–0.02]	**0.02** [0.02–0.02]	**0.02** [0.02–0.02]	−0.009 [−0.04, 0.02]	−0.006 [−0.08, 0.06]	−0.005 [−0.04, 0.03]	**2.2** [1.9–4.3]	**0.7** [0.3–1.7]	**0.8** [0.3–2.1]	−1.0 [−6, 4]	1.3 [−8, 11]	1.0 [−11, 13]
	ML1	0.56 ±0.13	0.60 ±0.11	0.59 ±0.13	0.04 [0.03–0.06]	0.04 [0.04–0.06]	0.04 [0.04–0.06]	**0.02** [0.02–0.08]	0.03 [0.02–0.05]	**0.02** [0.02–0.04]	0.019 [−0.14, 0.18]	0.003 [−0.08, 0.08]	0.008 [−0.72, 0.73]	57.1 [52.1–66.7]	49.8 [48.8–50.8]	49.8 [48.8–50.7]	−47.3 [−87, −8]	−54.9 [−76, −34]	−54.8 [−82, −28]
Forearm (*n* = 1964/830/980)	AO1	0.43 ±0.20	0.51 ±0.22	0.54 ±0.21	0.11 [0.08–0.13]	0.08 [0.06–0.12]	0.09 [0.07–0.12]	0.06 [0.03–0.08]	0.06 [0.04–0.09]	0.06 [0.04–0.08]	−0.002 [−0.48, 0.48]	0.000 [−0.38, 0.38]	0.000 [−0.45, 0.45]	11.1 [3.9–28.0]	7.1 [2.5–18.1]	5.5 [2.1–14.2]	−14.5 [−57, 28]	−9.6 [−48, 28]	−6.8 [−43, 29]
	TM1	0.67 ±0.14	0.63 ±0.19	0.61 ±0.22	0.08 [0.06–0.10]	0.10 [0.10–0.12]	0.10 [0.09–0.12]	0.04 [0.02–0.06]	0.06 [0.03–0.12]	0.06 [0.02–0.11]	−0.001 [−0.19, 0.18]	0.002 [−0.43, 0.43]	0.004 [−0.39, 0.39]	8.3 [2.2–18.2]	12.0 [5.4–22.7]	11.6 [5.4–21.1]	7.3 [−12, 27]	16.7 [−18, 51]	15.1 [−17, 47]
	RB1	0.70 ±0.16	0.77 ±0.16	0.76 ±0.17	0.10 [0.08–0.12]	0.07 [0.05–0.08]	0.08 [0.06–0.10]	0.05 [0.04–0.06]	0.06 [0.04–0.08]	0.06 [0.04–0.08]	0.009 [−0.26, 0.28]	0.023 [−0.30, 0.35]	0.023 [−0.42, 0.46]	13.6 [7.5–22.2]	10.6 [3.0–27.3]	10.7 [2.8–28.0]	−12.7 [−36, 10]	−18.9 [−63, 25]	−18.4 [−64, 27]
	RB2	0.88 ±0.11	0.80 ±0.19	0.80 ±0.20	**0.04** [0.04–0.06]	0.06 [0.04–0.10]	0.07 [0.04–0.10]	0.04 [0.04–0.06]	0.04 [0.02–0.06]	0.04 [0.02–0.06]	0.001 [−0.14, 0.14]	0.016 [−0.45, 0.48]	0.019 [−0.40, 0.44]	8.7 [4.3–15.8]	7.9 [2.0–23.4]	7.7 [1.7–23.7]	−8.7 [−28, 11]	−16.5 [−58, 25]	−16.9 [−62, 28]
	RB3	0.55 ±0.11	0.62 ±0.14	0.61 ±0.16	0.08 [0.08–0.10]	0.08 [0.06–0.10]	0.08 [0.06–0.10]	0.08 [0.06–0.12]	0.08 [0.05–0.10]	0.08 [0.05–0.12]	0.005 [−0.36, 0.37]	0.015 [−0.56, 0.59]	0.017 [−0.55, 0.58]	19.1 [9.6–30.5]	14.6 [6.9–30.4]	13.8 [6.0–28.6]	15.2 [−8, 39]	19.7 [−14, 53]	18.0 [−17, 53]
	FB1	0.77 ±0.20	0.64 ±0.23	0.62 ±0.27	0.10 [0.08–0.12]	0.12 [0.10–0.14]	0.12 [0.10–0.14]	0.04 [0.02–0.04]	0.04 [0.02–0.06]	0.03 [0.02–0.05]	0.013 [−0.22, 0.24]	0.017 [−0.36, 0.39]	0.013 [−0.49, 0.52]	9.1 [4.3–19.1]	14.8 [2.7–34.5]	5.4 [1.4–21.1]	−10.4 [−34, 13]	−21.4 [−63, 20]	−14.3 [−55, 26]
	FB2	0.87 ±0.17	0.73 ±0.26	0.77 ±0.25	**0.04** [0.03–0.06]	**0.05** [0.03–0.08]	0.05 [0.02–0.09]	0.06 [0.04–0.08]	0.06 [0.04–0.08]	0.06 [0.04–0.08]	0.018 [−0.19, 0.23]	0.032 [−0.49, 0.56]	0.026 [−0.30, 0.35]	8.7 [4.2–19.2]	15.5 [3.2–35.6]	5.7 [1.3–23.0]	−10.4 [−35, 14]	−22.0 [−64, 20]	−14.5 [−55, 26]
	FB3	**0.92** ±0.11	**0.90** ±0.16	**0.93** ±0.12	0.08 [0.06–0.10]	0.06 [0.03–0.10]	0.06 [0.04–0.08]	**0.02** [0.02–0.04]	**0.02** [0.02–0.03]	**0.02** [0.02–0.02]	−0.001 [−0.14, 0.13]	0.003 [−0.20, 0.21]	0.002 [−0.04, 0.04]	**3.5** [2.0–8.0]	**1.6** [0.7–4.0]	**1.4** [0.6–3.8]	0.1 [−22, 22]	−0.3 [−20, 20]	1.4 [−21, 24]
	ML1	0.05 ±0.08	0.16 ±0.18	0.17 ±0.20	**0.04** [0.02–0.06]	0.06 [0.04–0.08]	**0.04** [0.02–0.07]	0.06 [0.02–0.08]	0.04 [0.02–0.05]	0.04 [0.02–0.06]	−0.028 [−0.21, 0.15]	0.005 [−0.07, 0.08]	−0.004 [−0.15, 0.14]	98.0 [95.2–100.0]	49.8 [45.6–60.4]	49.3 [37.9–58.6]	−75.2 [−128, −22]	−52.7 [−95, −10]	−49.9 [−103, 3]
Head (*n* = 982/415/490)	AO1	0.26 ±0.15	0.37 ±0.20	0.39 ±0.20	0.14 [0.12–0.16]	0.14 [0.12–0.15]	0.12 [0.10–0.14]	**0.02** [0.02–0.04]	**0.02** [0.02–0.04]	**0.02** [0.02–0.05]	−0.003 [−0.12, 0.11]	−0.004 [−0.08, 0.07]	−0.004 [−0.09, 0.08]	5.0 [2.0–13.0]	3.1 [1.3–6.6]	2.8 [1.1–6.9]	−4.6 [−24, 14]	−1.7 [−23, 20]	−1.9 [−24, 20]
	TM1	0.75 ±0.13	0.76 ±0.15	0.78 ±0.14	**0.04** [0.02–0.06]	0.06 [0.04–0.08]	0.06 [0.04–0.08]	**0.02** [0.02–0.04]	0.03 [0.02–0.06]	**0.02** [0.02–0.06]	−0.003 [−0.04, 0.03]	0.004 [−0.05, 0.06]	0.004 [−0.06, 0.07]	7.1 [2.1–20.9]	13.1 [7.0–22.1]	14.2 [6.8–25.9]	9.0 [−19, 37]	18.5 [−16, 53]	19.4 [−15, 53]
	RB1	0.76 ±0.15	0.78 ±0.17	0.79 ±0.18	0.06 [0.04–0.08]	0.06 [0.04–0.08]	0.06 [0.04–0.07]	0.06 [0.04–0.09]	0.06 [0.04–0.10]	0.06 [0.04–0.10]	0.039 [−0.06, 0.14]	0.040 [−0.07, 0.16]	0.042 [−0.06, 0.15]	20.0 [10.0–32.0]	14.2 [4.0–28.9]	15.1 [4.1–34.1]	−18.2 [−48, 12]	−20.9 [−63, 22]	−22.3 [−73, 28]
	RB2	0.72 ±0.21	0.53 ±0.23	0.56 ±0.24	0.09 [0.06–0.12]	0.12 [0.10–0.14]	0.12 [0.10–0.14]	0.04 [0.03–0.06]	0.04 [0.02–0.06]	0.04 [0.02–0.06]	0.025 [−0.07, 0.12]	0.037 [−0.08, 0.16]	0.032 [−0.10, 0.16]	15.8 [8.9–27.1]	11.7 [3.3–28.4]	12.8 [2.7–30.6]	−15.7 [−43, 12]	−19.3 [−59, 20]	−21.0 [−65, 23]
	RB3	0.55 ±0.13	0.59 ±0.15	0.58 ±0.16	0.06 [0.05–0.08]	0.08 [0.06–0.10]	0.08 [0.06–0.10]	0.10 [0.06–0.12]	0.08 [0.06–0.12]	0.08 [0.05–0.12]	0.008 [−0.11, 0.13]	0.014 [−0.13, 0.15]	0.009 [−0.11, 0.13]	18.2 [7.7–30.4]	11.8 [4.4–24.9]	14.1 [5.2–29.1]	14.9 [−11, 41]	16.1 [−15, 47]	17.7 [−16, 51]
	FB1	**0.99** ±0.02	**0.97** ±0.08	0.95 ±0.10	**0.04** [0.02–0.06]	0.06 [0.04–0.08]	0.06 [0.04–0.08]	**0.02** [0.02–0.02]	**0.02** [0.02–0.02]	**0.02** [0.02–0.02]	0.000 [−0.03, 0.03]	0.008 [−0.03, 0.04]	0.010 [−0.02, 0.04]	2.3 [1.9–4.7]	1.1 [0.6–2.8]	**1.1** [0.5–3.0]	−2.0 [−6, 2]	−2.8 [−17, 11]	−3.2 [−22, 16]
	FB2	0.98 ±0.04	0.96 ±0.09	**0.97** ±0.06	0.06 [0.04–0.08]	**0.04** [0.02–0.06]	**0.04** [0.02–0.05]	**0.02** [0.02–0.02]	**0.02** [0.02–0.02]	**0.02** [0.02–0.02]	−0.001 [−0.03, 0.02]	0.007 [−0.03, 0.04]	0.009 [−0.03, 0.05]	2.3 [2.0–4.8]	1.2 [0.6–2.5]	1.2 [0.4–2.9]	−1.7 [−6, 3]	−2.3 [−16, 11]	−2.7 [−20, 14]
	FB3	0.98 ±0.05	**0.97** ±0.07	**0.97** ±0.07	**0.04** [0.04–0.06]	**0.04** [0.02–0.06]	**0.04** [0.02–0.04]	**0.02** [0.02–0.02]	**0.02** [0.02–0.02]	**0.02** [0.02–0.02]	−0.005 [−0.04, 0.03]	0.005 [−0.03, 0.05]	0.006 [−0.03, 0.05]	**2.2** [1.9–4.5]	**1.1** [0.5–2.5]	**1.1** [0.4–2.6]	0.1 [−15, 15]	−1.4 [−14, 11]	−2.0 [−17, 13]
	ML1	0.09 ±0.11	0.26 ±0.19	0.30 ±0.21	0.06 [0.04–0.09]	0.08 [0.04–0.12]	0.09 [0.04–0.12]	0.04 [0.02–0.09]	0.08 [0.04–0.10]	0.07 [0.04–0.10]	0.005 [−0.14, 0.15]	0.006 [−0.15, 0.16]	0.005 [−0.13, 0.14]	95.7 [91.3–100.0]	49.4 [34.3–57.0]	47.4 [28.9–52.5]	−73.7 [−130, −18]	−44.6 [−103, 14]	−41.3 [−103, 21]

**Table 6:** Sensor placements unique to CorLab dataset (Hand, Front Pocket, Back Pocket, Waist-Worn). There is one column per metric. $\tilde{F_{1}}$ is given as mean ± SD and the error metrics (${\tilde{\mathrm{MAE}}}_{\mathrm{IC}}$, ${\tilde{\mathrm{MAE}}}_{\mathrm{Step}}$ and ${\tilde{\%\mathrm{MRE}}}_{\mathrm{Cad}}$) as median [IQR]. The two BA-based columns (${\mathrm{MAE}}_{\mathrm{Step}}^{\mathrm{BA}}$ and ${\mathrm{Cad}}^{\mathrm{BA}}$) report agreement between predictions and ground truth as bias [LL,UL], where the bias is mean(Pred − GT) and LL, UL denote the lower and upper 95% limits of agreement. Best method(s) per column in **bold** (ties all shown); the BA groups are not bolded. *n* denotes the number of test executions.

Placement	Method	$\tilde{F_{1}}$	${\tilde{\mathrm{MAE}}}_{\mathrm{IC}}$ (s)	${\tilde{\mathrm{MAE}}}_{\mathrm{Step}}$ (s)	${\mathrm{MAE}}_{\mathrm{Step}}^{\mathrm{BA}}$ (s)	${\tilde{\%\mathrm{MRE}}}_{\mathrm{Cad}}$	${\mathrm{Cad}}^{\mathrm{BA}}$ (spm)
Hand (*n* = 1964)	AO1	0.45 ±0.19	0.10 [0.08–0.12]	0.06 [0.04–0.09]	−0.004 [−0.50, 0.50]	13.3 [4.3–33.3]	−16.2 [−59, 27]
	TM1	0.65 ±0.16	0.08 [0.06–0.10]	0.04 [0.03–0.07]	0.001 [−0.23, 0.23]	7.4 [2.2–16.0]	6.6 [−11, 24]
	RB1	0.66 ±0.17	0.10 [0.08–0.12]	0.06 [0.04–0.08]	0.011 [−0.35, 0.37]	13.6 [7.5–22.2]	−12.7 [−35, 10]
	RB2	0.88 ±0.11	**0.04** [0.04–0.06]	0.04 [0.04–0.06]	0.003 [−0.12, 0.12]	9.1 [4.5–16.7]	−9.2 [−30, 11]
	RB3	0.54 ±0.11	0.08 [0.08–0.10]	0.09 [0.06–0.12]	0.005 [−0.41, 0.42]	19.4 [9.6–30.4]	15.1 [−8, 38]
	FB1	0.73 ±0.20	0.10 [0.08–0.12]	0.04 [0.02–0.06]	0.015 [−0.28, 0.31]	10.5 [4.5–21.1]	−11.4 [−35, 13]
	FB2	0.85 ±0.17	**0.04** [0.04–0.06]	0.06 [0.04–0.08]	0.022 [−0.18, 0.22]	10.6 [4.8–23.4]	−11.8 [−37, 14]
	FB3	**0.93** ±0.10	0.06 [0.04–0.10]	**0.02** [0.02–0.04]	−0.002 [−0.15, 0.14]	**3.5** [2.0–8.0]	−0.5 [−20, 19]
	ML1	0.04 ±0.06	0.06 [0.04–0.10]	0.08 [0.04–0.16]	0.013 [−0.22, 0.24]	98.0 [95.5–100.0]	−75.4 [−128, −23]
Front Pocket (*n* = 993)	AO1	0.29 ±0.13	0.13 [0.12–0.14]	0.04 [0.02–0.10]	0.001 [−0.60, 0.60]	8.3 [3.9–15.8]	−4.9 [−25, 15]
	TM1	0.72 ±0.11	0.05 [0.04–0.06]	0.06 [0.04–0.09]	−0.003 [−0.22, 0.21]	17.4 [4.3–31.8]	14.0 [−14, 42]
	RB1	0.71 ±0.27	0.08 [0.06–0.10]	0.12 [0.06–0.16]	0.028 [−0.30, 0.35]	16.7 [6.2–36.4]	−17.4 [−51, 16]
	RB2	0.80 ±0.16	0.08 [0.06–0.10]	0.04 [0.02–0.06]	0.013 [−0.27, 0.30]	12.5 [4.3–31.6]	−14.8 [−46, 17]
	RB3	0.63 ±0.12	0.07 [0.06–0.08]	0.10 [0.06–0.12]	0.001 [−0.31, 0.31]	31.9 [18.0–43.2]	23.0 [−5, 51]
	FB1	**0.99** ±0.02	0.04 [0.02–0.06]	**0.02** [0.02–0.04]	−0.001 [−0.08, 0.08]	2.3 [2.0–4.5]	−1.9 [−6, 2]
	FB2	0.98 ±0.04	0.06 [0.04–0.08]	**0.02** [0.02–0.04]	−0.002 [−0.13, 0.12]	2.3 [2.0–4.5]	−1.6 [−7, 3]
	FB3	0.98 ±0.03	**0.02** [0.02–0.04]	**0.02** [0.02–0.02]	−0.008 [−0.03, 0.02]	**2.2** [0.0–4.3]	−0.5 [−8, 7]
	ML1	0.29 ±0.20	0.06 [0.03–0.09]	0.07 [0.03–0.12]	−0.000 [−0.17, 0.17]	82.3 [68.2–93.5]	−62.4 [−114, −11]
Back Pocket (*n* = 960)	AO1	0.44 ±0.16	0.10 [0.08–0.12]	0.12 [0.06–0.18]	0.002 [−0.55, 0.56]	16.5 [6.0–43.2]	9.6 [−32, 52]
	TM1	0.65 ±0.11	0.05 [0.04–0.07]	0.08 [0.04–0.12]	0.007 [−0.29, 0.30]	39.1 [19.7–50.0]	27.2 [−8, 62]
	RB1	0.86 ±0.13	0.04 [0.04–0.06]	0.08 [0.06–0.12]	0.032 [−0.25, 0.31]	14.0 [5.3–30.4]	−14.8 [−45, 16]
	RB2	0.62 ±0.25	0.12 [0.10–0.14]	0.04 [0.02–0.06]	0.010 [−0.35, 0.37]	8.7 [4.3–18.8]	−10.8 [−37, 15]
	RB3	0.66 ±0.12	0.06 [0.06–0.08]	0.08 [0.06–0.12]	0.001 [−0.33, 0.34]	28.0 [15.8–38.8]	21.4 [−5, 47]
	FB1	0.83 ±0.19	0.06 [0.04–0.08]	0.08 [0.06–0.10]	0.020 [−0.18, 0.22]	4.0 [2.1–7.0]	−3.1 [−12, 6]
	FB2	0.95 ±0.08	0.04 [0.04–0.06]	0.08 [0.04–0.12]	0.015 [−0.13, 0.16]	3.9 [2.0–6.5]	−2.4 [−11, 6]
	FB3	**0.98** ±0.06	**0.02** [0.02–0.04]	**0.02** [0.02–0.02]	−0.003 [−0.03, 0.03]	**2.2** [0.0–4.5]	−0.7 [−9, 8]
	ML1	0.20 ±0.20	0.10 [0.06–0.12]	0.06 [0.04–0.12]	0.006 [−0.36, 0.37]	80.0 [66.0–91.6]	−61.2 [−114, −9]
Waist-Worn (*n* = 993)	AO1	0.28 ±0.16	0.14 [0.12–0.16]	**0.02** [0.02–0.04]	−0.005 [−0.49, 0.48]	6.5 [2.2–15.0]	−5.5 [−23, 12]
	TM1	0.68 ±0.14	**0.04** [0.04–0.06]	0.04 [0.02–0.06]	−0.004 [−0.21, 0.20]	10.7 [2.1–25.6]	10.5 [−18, 39]
	RB1	0.86 ±0.19	0.06 [0.04–0.08]	0.06 [0.04–0.08]	0.019 [−0.16, 0.20]	7.7 [4.1–16.7]	−9.9 [−36, 16]
	RB2	0.85 ±0.18	0.10 [0.07–0.12]	0.03 [0.02–0.04]	0.013 [−0.12, 0.15]	5.0 [2.4–12.5]	−8.3 [−32, 16]
	RB3	0.64 ±0.17	0.06 [0.05–0.08]	0.06 [0.04–0.09]	−0.003 [−0.33, 0.32]	15.6 [6.2–30.0]	14.0 [−13, 41]
	FB1	**0.99** ±0.02	**0.04** [0.02–0.05]	**0.02** [0.02–0.02]	−0.002 [−0.08, 0.08]	**2.2** [1.9–4.5]	−1.8 [−6, 2]
	FB2	0.97 ±0.06	0.06 [0.04–0.08]	**0.02** [0.02–0.02]	−0.003 [−0.05, 0.04]	2.3 [2.0–4.5]	−1.6 [−6, 3]
	FB3	0.98 ±0.04	0.06 [0.04–0.08]	**0.02** [0.02–0.02]	−0.006 [−0.03, 0.02]	**2.2** [1.9–4.3]	−1.2 [−6, 4]
	ML1	0.19 ±0.18	0.06 [0.04–0.10]	0.06 [0.02–0.10]	−0.005 [−0.43, 0.42]	91.3 [79.0–96.3]	−68.2 [−125, −12]

*Sensor placements shared by the CorLab and WearGait-PD datasets (Mid-Upper Leg, Forearm, Head).* At the remaining placements shared by CorLab and WearGait-PD (Table 5), FB3 remained robust ($\tilde{F_{1}}\geq 0.9$), whereas FB1 and FB2 degraded markedly at the distal Forearm (∼0.62–0.87) while remaining strong at the Head (≥0.95). ML1 showed the steepest dependence on placement of all the evaluated methods: it reached an $\tilde{F_{1}}$ of 0.56–0.60 at the Mid-Upper Leg, comparable to that of RB1, but fell to 0.09–0.30 at the Head and to 0.05–0.17 at the Forearm, the lowest values recorded at those two locations.

Detection completeness did not always coincide with timing accuracy: TM1, RB1, FB2, and FB3 timed the matched initial contacts to within ${\tilde{\mathrm{MAE}}}_{\mathrm{IC}}\leq 0.10$ s even when their $\tilde{F_{1}}$ was lower, indicating that their lower $\tilde{F_{1}}$ values arose from missed or spurious detections rather than from imprecise timing. The ${\tilde{\%\mathrm{MRE}}}_{\mathrm{Cad}}$ increased specifically at the Forearm for FB1 and FB2. FB3 again produced the smallest ${\tilde{\mathrm{MAE}}}_{\mathrm{Step}}$ (≈0.02 s) and the lowest ${\tilde{\%\mathrm{MRE}}}_{\mathrm{Cad}}$ (e.g., in WearGait-PD, 0.68–1.57% for HC and 0.77–1.42% for PD), whereas TM1 and the rule-based methods reached up to ∼43%.

The agreement analysis reflected the same placement sensitivity: at the Forearm the cadence limits widened sharply for FB1 (bias −21.4 steps/min in WearGait HC, with wide limits), while FB3 stayed close to zero and retained a near-zero step-duration bias throughout.

*Sensor placements unique to CorLab dataset (Hand, Front Pocket, Back Pocket, Waist-Worn).* At the placements unique to CorLab (Table 6), FB3 remained among the most robust, reaching an $\tilde{F_{1}}$ of 0.93 at the hand and 0.98 at both pocket placements and the waist-worn bag. FB1 and FB2 were also strong at the front pocket and waist-worn bag ($\tilde{F_{1}}$ of 0.97–0.99) but degraded at the hand (FB1 0.73, FB2 0.85), with FB1 also falling to 0.83 at the back pocket, confirming the hand as the most difficult placement. AO1 was again the weakest method ($\tilde{F_{1}}$ of 0.28–0.45), while the rule-based methods were the most sensitive to placement: RB1 held up at the more proximal waist-worn and back-pocket positions (0.86) but fell at the front pocket and hand (0.66–0.71). TM1 and RB3 also returned comparatively low $\tilde{F_{1}}$ values. ML1 recorded the lowest $\tilde{F_{1}}$ at all four of these placements, ranging from 0.29 at the front pocket to 0.04 at the hand, the poorest result of any method at any placement in the benchmark.

FB3 also produced the smallest ${\tilde{\mathrm{MAE}}}_{\mathrm{Step}}$ (0.02 s) and the lowest ${\tilde{\%\mathrm{MRE}}}_{\mathrm{Cad}}$ (2.2–3.5%) at every location, whereas TM1 and RB3 reached markedly higher ${\tilde{\%\mathrm{MRE}}}_{\mathrm{Cad}}$ values (up to 39.1% and 31.9%, respectively).

The Bland–Altman analysis showed that the three evaluated frequency-based algorithms held near-zero step-duration and cadence biases with the narrowest limits at every location (e.g., FB3 cadence bias of −0.5 steps/min at the front pocket), whereas TM1 and RB3 overestimated cadence, with biases up to +27.2 and +21.4 steps/min at the back pocket.

*Error decomposition by placement, cohort, and environment.* Step-detection errors can arise from three distinct mechanisms: a reference initial contact may be missed (FN), a spurious contact may be detected (FP), or a correctly detected contact (i.e., a predicted IC that falls within an ${\mathrm{IC}}_{\mathrm{REF}}$ window) may be assigned an incorrect timing. To investigate how these error mechanisms contribute to algorithm performance under different conditions, Figure 1 decomposes the results according to the three factors considered in the benchmark design: sensor placement, clinical cohort, and recording environment.

Sensor placement affected the algorithms to different degrees. The upper-left panel compares recall and precision across the eight placements. FB2 and FB3 maintained high precision throughout, but FB3 had more consistent recall (0.93–1.00, compared with 0.78–0.99 for FB2). Six of the nine algorithms recorded their lowest recall at the forearm or hand. Missed contacts accounted for most errors in ML1, RB1, and RB2. ML1 showed this pattern most clearly: despite relatively high precision, it missed most reference contacts. By contrast, RB3 and TM1 had lower precision than recall, with false positives accounting for most errors. AO1 performed poorly on both measures.

Detection quality and timing accuracy did not always follow the same pattern (middle-left panel). FB2 and FB3 combined high $F_{1}$ values with timing errors of 30–70 ms. Timing errors varied more with placement for FB1 and RB2, while AO1 had relatively large errors throughout (99–133 ms). ML1 illustrated why the two metrics should be considered together: its $F_{1}$ fell as low as 0.04, yet its timing errors across placements remained between 47 and 96 ms. These timing estimates described only the matched contacts, even when many reference contacts were missed.

The lower-left panel shows the proportion of executions contributing to the timing estimates. Contact coverage exceeded 93% at every placement for all methods except ML1. Step coverage was lower because it required two consecutive matched contacts. FB2 and FB3 maintained contact coverage above 99% and step coverage of at least 94.5%. Step coverage varied more for RB1 and RB2. For ML1, it remained below one third of the executions at every placement, limiting the recordings represented by its ${\mathrm{MAE}}_{\mathrm{Step}}$ estimates.

Similar error patterns appeared across clinical cohorts (upper-centre panel). FB2 and FB3 maintained recall above 0.90 in all six cohorts. ML1 again combined high precision with low recall, while RB3 and TM1 had higher recall than precision. RB1 and RB2 showed the largest variation in recall between cohorts, with ranges spanning 0.24 and 0.36, respectively. FB2 and FB3 also combined high $F_{1}$ values with low timing errors across cohorts (middle-centre panel). RB2 varied the most on both measures: $F_{1}$ ranged from 0.41 to 0.78, and ${\mathrm{MAE}}_{\mathrm{IC}}$ from 74 to 127 ms. ML1 had timing errors comparable to those of FB2 and FB3, despite substantially lower $F_{1}$ values.

Contact coverage remained high for FB1, FB2, FB3, RB1, RB3, and TM1 (lower-centre panel). Coverage was lower for RB2 and ML1. In the COPD cohort, RB2 had 85% contact coverage and 50% step coverage. ML1’s step coverage ranged from 10% to 43% across cohorts. These comparisons pooled different combinations of datasets and sensor placements, so the differences cannot be attributed to clinical condition alone.

The pooled comparison between recording environments showed changes in both directions (upper-right panel). RB1 had higher precision and recall during free-living walking, whereas RB2 and RB3 had lower precision. FB2 and FB3 maintained high values for both measures, while ML1 continued to miss most reference contacts. Mean $F_{1}$ was lower during free-living walking for eight of the nine algorithms, with RB1 the only exception (middle-right panel). RB2 had the largest reduction (0.28). FB2 and FB3 remained the strongest methods in this setting, with mean $F_{1}$ values of 0.87 and 0.85, respectively, and timing errors of 43–55 ms across the two environments. Timing errors increased for RB2 and TM1 but decreased for RB1.

Contact coverage remained close to complete in both environments for FB1, FB2, FB3, RB1, RB3, and TM1, with step coverage above 86% (lower-right panel). AO1 and RB2 showed larger reductions in both measures during free-living walking. ML1 had the lowest contact coverage in both environments, although its step coverage was higher during free-living walking (28%) than in the laboratory (11%). The laboratory grouping included eight sensor placements, while the free-living grouping included only the lower back. These results therefore describe the two pooled recording conditions rather than an isolated effect of recording environment.

*Statistical comparison of the algorithms.* The Friedman test rejected the null hypothesis of equal average ranks for all four criteria (see Table 7). Rankings showed substantial concordance across evaluation blocks for $\tilde{F_{1}}$ (W=0.785), ${\tilde{\mathrm{MAE}}}_{\mathrm{Step}}^{\mathrm{corr}}$ (W=0.734), and ${\tilde{\%\mathrm{MRE}}}_{\mathrm{Cad}}$ (W=0.67), whereas ${\tilde{\mathrm{MAE}}}_{\mathrm{IC}}^{\mathrm{corr}}$ showed moderate concordance (W=0.547). Thus, the relative ordering of the algorithms was generally stable across evaluation blocks, with greater variation observed for ${\tilde{\mathrm{MAE}}}_{\mathrm{IC}}^{\mathrm{corr}}$.

A consistent pattern was observed for the frequency-based methods: FB1, FB2, and FB3 occupied the three highest average ranks for $\tilde{F_{1}}$, ${\tilde{\mathrm{MAE}}}_{\mathrm{Step}}^{\mathrm{corr}}$, and ${\tilde{\%\mathrm{MRE}}}_{\mathrm{Cad}}$, although their relative ordering differed across criteria. For ${\tilde{\%\mathrm{MRE}}}_{\mathrm{Cad}}$, FB1 ranked first, followed by FB3 and FB2, indicating particularly strong performance of these three algorithms for cadence estimation.

FB2 and FB3 were the only algorithms that were not significantly outperformed by another method for any of the four criteria. Depending on the criterion, the Nemenyi test identified significant differences in 19–22 of the 36 pairwise comparisons; the remaining comparisons should be considered unresolved rather than evidence of equivalence. Overall, these results indicated that, under the conditions evaluated in this benchmark, the tested frequency-based implementations tended to achieve better average ranks than the other tested algorithms. This observation concerns the specific implementations evaluated here and should not be interpreted as evidence that frequency-based methods as a family are generally superior.

**Table 7:** Average Friedman ranks (1= best) of the nine algorithms over N=37 evaluation blocks, with Nemenyi post hoc groupings and omnibus statistics. The best rank in each column is shown in **bold**. Within a column, values sharing a superscript letter are not significantly different at α=0.05 (Nemenyi test, critical difference CD=1.97); values sharing no letter differ significantly. *W* denotes Kendall’s coefficient of concordance. All four omnibus tests rejected the null hypothesis of equal average ranks.

Algorithm	$\tilde{F_{1}}$	${\tilde{\mathrm{MAE}}}_{\mathrm{IC}}^{\mathrm{corr}}$	${\tilde{\mathrm{MAE}}}_{\mathrm{Step}}^{\mathrm{corr}}$	${\tilde{\%\mathrm{MRE}}}_{\mathrm{Cad}}$
FB3	**1.86** ^a^	2.14 ^a^	**1.24** ^a^	2.41 ^a^
FB2	1.89 ^a^	**1.86** ^a^	2.65 ^ab^	3.03 ^ab^
FB1	3.46 ^ab^	6.05 ^b^	3.70 ^bc^	**2.38** ^a^
RB2	5.00 ^bc^	6.30 ^bc^	5.05 ^cd^	4.16 ^abc^
TM1	5.35 ^bc^	4.57 ^b^	4.32 ^bc^	6.76 ^def^
RB1	4.35 ^bc^	4.51 ^b^	5.68 ^cd^	5.41 ^cde^
ML1	8.59 ^e^	5.30 ^b^	9.00 ^e^	8.65 ^f^
AO1	8.16 ^de^	8.22 ^c^	6.97 ^d^	4.89 ^bcd^
RB3	6.32 ^cd^	6.05 ^b^	6.38 ^d^	7.32 ^ef^
$\chi_{F}^{2}$	232.27	161.80	217.23	198.21
*p*	$9.82\times 10^{-46}$	$6.72\times 10^{-31}$	$1.48\times 10^{-42}$	$1.53\times 10^{-38}$
*W*	0.785	0.547	0.734	0.670

### 3.5. Discussion

The benchmark indicates that sensor placement and recording environment were more influential than clinical cohort in shaping algorithm performance. Performance was generally strongest at proximal sensor placements and under controlled laboratory conditions, whereas distal placements and free-living recordings posed the greatest challenges, consistent with previous studies [3,24]. By comparison, performance varied relatively little across clinical cohorts for most methods. Within the conditions represented in this benchmark, these findings suggest that algorithm design and measurement context had a greater influence on performance than gait pathology alone. The following discussion considers the effects of sensor placement, pathological gait and recording environment.

*The Impact of Sensor Placement.* Consistent with prior literature, sensor placement affected algorithm performance in different ways. Placements near the centre of mass, such as the lower back, generally provided the most reliable step-detection performance. By contrast, at distal placements, particularly the hand and forearm, differences between algorithms became more pronounced. FB3 showed the most consistent performance across sensor placements, whereas the remaining methods were more sensitive to changes in sensor location.

The error decomposition indicates that these differences arose from different sources. For FB1, FB2, RB1, and RB2, the errors resulted primarily from a loss of sensitivity, as most of their errors were missed contacts. This pattern is consistent with distal-limb acceleration signals not always preserving the shape or frequency content used by these methods. By contrast, RB3 and TM1 produced more false-positive detections, suggesting that arm movements unrelated to gait could satisfy their criteria for identifying a point as an initial contact. Performance at distal placements can therefore deteriorate through either missed contacts or false-positive detections, depending on the algorithm.

The three evaluated frequency-based algorithms consistently achieved the highest average rankings across metrics. FB3 demonstrated the greatest resilience at distal locations, which may be attributed to its adaptive frequency selection. At proximal and more constrained placements, however, no significant differences were observed among the three frequency-based methods. However, the robustness of the three evaluated frequency-based algorithms across sensor placements should be interpreted within the scope of the present evaluation framework. Their performance may depend on the specific sensor placement and experimental conditions, and these findings should therefore not be interpreted as evidence of a general superiority.

*Robustness to Pathological Gait in Structured Environments.* In the structured walking tests from the WearGait-PD and Mobilise-D datasets, FB1, FB2, and FB3 maintained similar step-detection performance across pathological cohorts and healthy controls. This suggests that their performance generalised well across the gait patterns represented in these tests.

By contrast, AO1, RB2, and RB3 showed lower recall than FB1, FB2 and FB3 in both healthy and pathological cohorts. Their lower performance therefore appears to reflect a general loss of sensitivity rather than a specific failure when applied to pathological gait. RB1 and RB2 showed greater variation between cohorts, which may reflect the sensitivity of their fixed peak-prominence criteria to changes in signal morphology. Overall, the results suggested that algorithm design had a greater influence on step-detection performance than the presence of gait pathology alone.

*The Challenge of Free-Living Environments.* The transition from controlled laboratory tests to unsupervised real-world recordings posed a major challenge for step detection. Most methods showed lower performance in the free-living grouping than in the laboratory grouping. FB2 and FB3 remained among the strongest-performing methods, although their performance also decreased. RB1 showed the opposite pattern; however, this comparison requires caution because the laboratory grouping included distal placements, whereas the free-living recordings were collected only at the lower back. The differences between these groupings can therefore not be attributed to the recording environment alone.

The type of error also depended on the algorithm. Methods prone to false-positive detections tended to overestimate cadence, whereas those that missed contacts tended to underestimate it. Changes in walking speed, brief pauses, and irregular movements may alter the signal patterns on which these methods rely. Consequently, some algorithms may identify movements unrelated to gait as initial contacts, whereas others may fail to recognise true contacts when the signal characteristics change.

These factors were not analysed separately in the benchmark, and turns were excluded from the evaluation. Nevertheless, the results indicate that good performance under laboratory conditions does not necessarily translate to free-living walking. Reliable step detection from a single IMU therefore remains an open challenge for longitudinal monitoring in unconstrained environments.

*Limitations.* While the benchmark comparison covered three large datasets encompassing multiple sensor placements, diverse clinical cohorts, and both controlled and free-living conditions, some limitations should be acknowledged. First, the benchmark was restricted to nine step detection algorithms and did not cover the full breadth of existing step detection methods. Of the six families reviewed in Section 2, five are represented experimentally; the phase portrait family is not, so its characterisation in Section 2.2 rests on the published literature, and the conclusions drawn from the benchmark do not extend to it. Second, the machine learning family is represented by a single pre-trained model, applied without adaptation and originally developed for a shank-worn sensor; its performance at the remaining placements therefore reflects transferability rather than the attainable performance of learning-based methods in general. Third, the Mobilise-D dataset provided a single IMU placement (lower back), precluding sensor placement comparisons for free-living conditions. Fourth, ground-truth collection methods differed across datasets, which may introduce subtle inconsistencies in the reference standards. Although the algorithms were compared within each evaluation block, differences between reference systems may still contribute to differences in absolute error magnitudes across datasets and should therefore be considered when interpreting cross-dataset comparisons. Fifth, although all algorithms were run on the complete recordings, reference events and algorithm detections occurring within identified turn windows were excluded from the performance evaluation. The benchmark therefore characterised step-detection performance during uninterrupted straight-line walking but did not establish performance during turns or other complex locomotor behaviours. This restriction limits the generalisability of the results to real-world mobility, where performance during changes in walking direction and other locomotor transitions remains unassesed. Finally, all algorithms were evaluated offline; real-time implementation constraints, such as latency, computational cost, and suitability for deployment on resource-constrained embedded devices, were not assessed.

## 4. Conclusions

Selecting an appropriate step detection algorithm for longitudinal motor assessment requires both a theoretical understanding of available methods and empirical evidence of their performance across realistic deployment conditions. First, a structured taxonomic review of IMU-based step detection methods was presented, organising the landscape into six categories—rule-based heuristics, phase portrait, template matching, time–frequency analysis, adaptive oscillators, and machine learning— and characterising the trade-offs of each. On the basis of the published literature, the review indicated that computational efficiency and interpretability come at the cost of generalisability in rule-based and phase portrait methods, that template matching is inherently constrained by its dependence on pre-segmented templates and cold-start requirements, and that time–frequency methods offer the most favourable balance between noise robustness and adaptability across diverse gait conditions.

Second, an extensive benchmark comparison across three datasets confirmed that step detection in structured laboratory settings is tractable, even across pathological cohorts and a wide range of sensor placements. Free-living monitoring, however, remains a significant open challenge: the transition from controlled to unsupervised environments can introduce systematic performance degradation driven by false-positive detections from non-gait activities, environmental variability, and unconstrained device placement. Among the method families represented in the benchmark, the three evaluated frequency-based methods showed the most consistently favourable performance across the conditions tested. However, these findings are limited to the implementations evaluated in this study and to the specific sensor placements, cohorts, and recording environments considered. They should therefore not be interpreted as evidence of general superiority of any particular method family, and the applicability and relative performance of different approaches should be assessed case by case according to the characteristics of the target application.

## Figures and Tables

**Figure 1:**
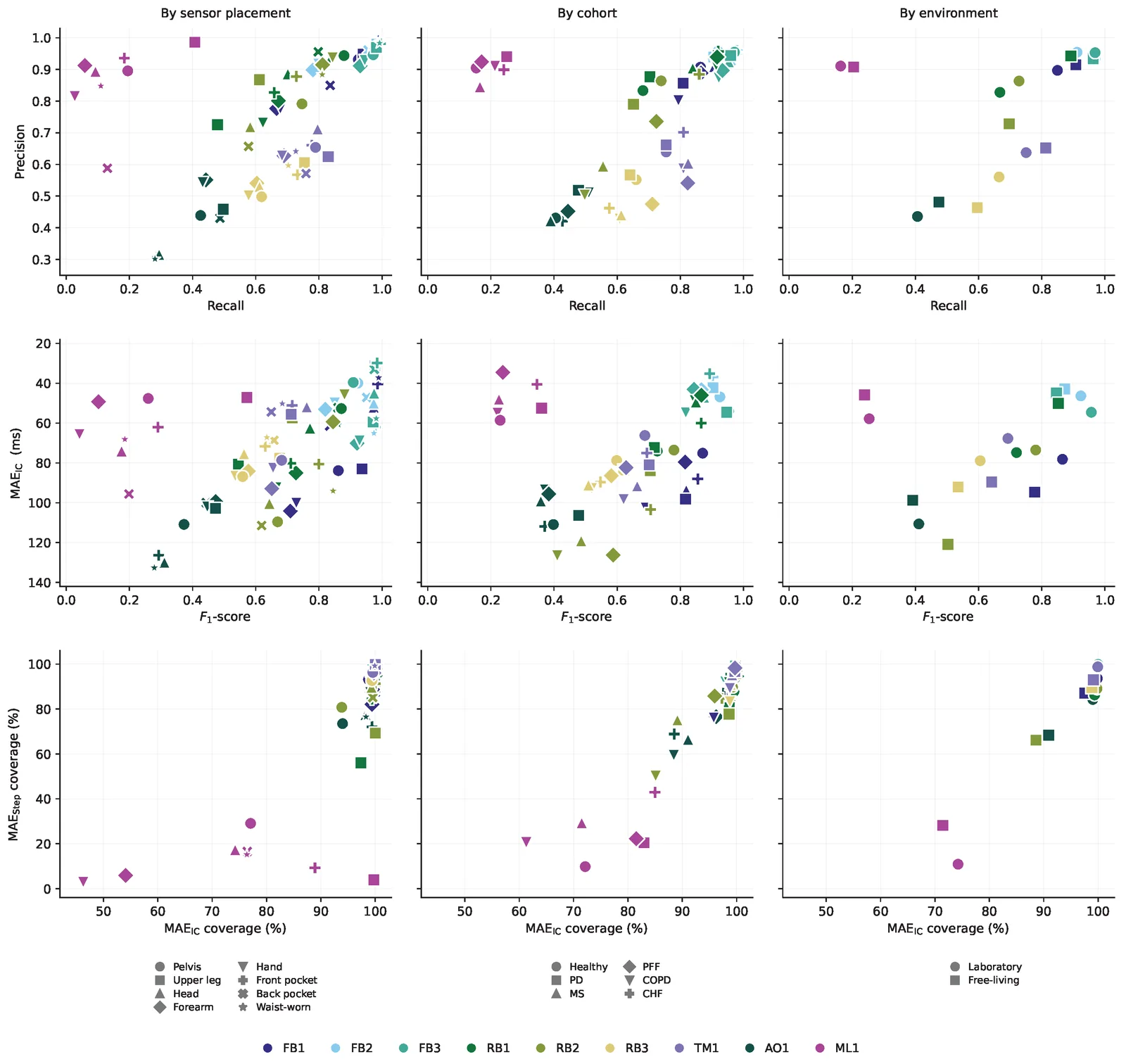
Error decomposition of step-detection performance (top), detection quality versus timing accuracy (middle), and coverage of the two timing-error metrics (bottom) for the nine algorithms across sensor placement, clinical cohort, and recording environment. Each point represents one algorithm under one condition, pooled over all executions meeting that condition. In the top row, precision and recall are micro-averaged from the summed TP, FP, and FN counts across all executions meeting the corresponding condition. In the middle row, *F*_1_-score and MAEIC are averaged across executions for each condition. The bottom row shows the percentage of executions for which MAEIC and ${\mathrm{MAE}}_{\mathrm{Step}}$ could be calculated for each condition. Colours identify algorithms and marker shapes identify conditions. The vertical axis of the middle row is inverted so that better results are consistently higher.

**Table 1:** Taxonomy of Step Detection Methods. A comparative summary of operational advantages and limitations, synthesised from [3,7] and the references discussed in Section 2.

Method Family	Key Advantages	Key Limitations
Rule-Based (Time-Domain)	Interpretable ‘White-box’ logic High computational efficiency Ideal for real-time/embedded systems	High parametric sensitivity Filter phase lag (latency) Sensor location dependent
Phase Portrait (Geometric)	Continuous gait phase info Computationally light Single-sensor effective	Assumes geometric regularity Fails in transient/abnormal gait Requires stable phase locking
Template Matching (Pattern Recognition)	Handles morphological variability Highly customizable Robust to speed changes	High computational cost Cold start (needs template) Pre-segmentation required
Frequency-Based (Spectral)	Intrinsic noise robustness Multiresolution Analysis Simultaneous denoising & detection	Time–Frequency trade-off Sensitive to Mother Wavelet choice High complexity (time–frequency representation enhancement)
Adaptive Oscillators (Dynamical Systems)	Smooth continuous estimation No filtering phase-lag Sensor agnostic	Convergence latency (start/stop) Tuning trade-off (stability vs. reactivity) Struggles with asymmetry
Machine Learning (Statistical/Data-Driven)	Automated feature extraction Independence from handcrafted thresholds and peak-detection heuristics Successful modeling of complex gait and temporal dependencies	Data hungry (large labeled datasets) Limited explainability High computational load Vulnerable to domain shifts across sensor placements, devices, and cohorts.

**Table 2:** Selected step-detection algorithms and implementation sources. RB1, RB2, FB1, and FB2 were executed using MobGap v0.4.0 with its default configuration. The non-machine-learning methods were run in Python 3.9.6. ML1 inference was performed in a Python 3.11.15 environment using TensorFlow 2.21.0 to satisfy the dependency requirements of the authors’ published code. All computations were performed offline on a MacBook Pro equipped with an Apple M2 Pro processor (10-core CPU) and 16 GB of unified memory.

Method Family	Algorithm	Implementation	Acronym
Rule-Based	Shin and Park (2011) [18]	MobGap Library ^1^	RB1
	Lee et al. (2010) [61]	MobGap Library ^1^	RB2
	Benson et al. (2019) [62]	Adapted from the original description of the method	RB3
Frequency-Based	McCamley et al. (2012) [37]	MobGap Library ^1^	FB1
	Ionescu et al. (2020) [6]	MobGap Library ^1^	FB2
	Ena et al. (2024, 2026) [41,42]	Adapted from the original description of the method	FB3
Template Matching	Filippou et al. (2023) [63]	Adapted from authors’ code ^2^	TM1
Adaptive Oscillators	Tomc and Matjačić (2023) [50]	Adapted from the original description of the method	AO1
Machine Learning	Romijnders et al. (2022) [10]	Pre-trained weights from the authors’ original code ^3^	ML1

^1^ https://github.com/mobilise-d/mobgap (accessed on 24 March 2026). ^2^ https://github.com/ValeriaF22/Thesis-Project (accessed on 28 January 2026). ^3^ https://github.com/rmndrs89/my-gait-events-tcn (accessed on 29 January 2026).

**Table 3:** Summary of participant demographics and clinical characteristics (mean ± standard deviation) across the three benchmark datasets. Abbreviations: HC = Healthy Controls; PD = Parkinson’s Disease; MS = Multiple Sclerosis; PFF = Proximal Femoral Fracture; COPD = Chronic Obstructive Pulmonary Disease; CHF = Congestive Heart Failure; EDSS = Expanded Disability Status Scale; UPDRS III = Unified Parkinson’s Disease Rating Scale (Motor subscale); H&Y = Hoehn and Yahr scale; SPPB = Short Physical Performance Battery; CAT = COPD Assessment Test; KCCQ = Kansas City Cardiomyopathy Questionnaire. *n* denotes the number of participants.

Dataset		HC	PD	MS	PFF	COPD	CHF
CorLab	*n*, Sex (M/F)	83 (38/45)	-	-	-	-	-
	Age (years)	42.84±15.7	-	-	-	-	-
	Height (m)	1.68±0.82	-	-	-	-	-
	Weight (kg)	73.2±15.6	-	-	-	-	-
Mobilise-D	*n*, Sex (M/F)	20 (11/9)	20 (16/4)	20 (11/9)	19 (8/11)	17 (9/8)	12 (8/4)
	Age (years)	71.7±5.8	69.8±7.2	48.7±9.7	80.0±8.5	69.4±9.1	69.1±11.7
	Height (m)	1.66±0.10	1.73±0.07	1.71±0.13	1.69±0.08	1.69±0.07	1.74±0.10
	Weight (kg)	75.1±11.8	78.2±14.4	84.0±22.9	68.4±16.0	73.7±14.2	84.5±16.8
	EDSS	-	-	3.5±1.7	-	-	-
	UPDRS III	-	28.4±13.6	-	-	-	-
	H&Y score	-	2.05±0.69	-	-	-	-
	SPPB	-	-	-	6.2±3.9	-	-
	CAT score	-	-	-	-	16.6±8.9	-
	KCCQ	-	-	-	-	-	80.5±20.2
WearGait-PD	*n*, Sex (M/F)	85 (38/47)	100 (35/65)	-	-	-	-
	Age (years)	74.1±9.2	68.6±4.1	-	-	-	-
	Height (m)	1.69±0.13	1.74±0.10	-	-	-	-
	Weight (kg)	80.6±16.9	78.1±17.3	-	-	-	-
	UPDRS III	-	25±10	-	-	-	-
	H&Y score	-	2.15±0.61	-	-	-	-

## Data Availability

This study analysed three datasets. The WearGait-PD dataset is openly available [11]. The Mobilise-D Technical Validation Study dataset is available on Zenodo at https://doi.org/10.5281/zenodo.15861907. The CorLab dataset is not publicly available due to privacy and ethical restrictions but could be available from the corresponding author on reasonable request.
